# mir-101-3p Downregulation Promotes Fibrogenesis by Facilitating Hepatic Stellate Cell Transdifferentiation During Insulin Resistance

**DOI:** 10.3390/nu11112597

**Published:** 2019-10-29

**Authors:** Marica Meroni, Miriam Longo, Veronica Erconi, Luca Valenti, Stefano Gatti, Anna Ludovica Fracanzani, Paola Dongiovanni

**Affiliations:** 1 General Medicine and Metabolic Diseases, Fondazione IRCCS Ca’ Granda Ospedale Maggiore Policlinico, Milano 20122, Italy; maricameroni11@gmail.com (M.M.); longo.miriam92@gmail.com (M.L.); veronica.erconi@studenti.unimi.it (V.E.); anna.fracanzani@unimi.it (A.L.F.); 2 Department of Pathophysiology and Transplantation, Università degli Studi di Milano, Milano 20122, Italy; 3 Department of Clinical Sciences and Community Health, Università degli Studi di Milano, Milano 20122, Italy; 4 Transfusional Center–Translational Medicine, Fondazione IRCCS Cà Granda Ospedale Maggiore Policlinico Milano, Milano 20122, Italy; luca.valenti@unimi.it; 5 Preclinical Research Center, Fondazione IRCCS Ca’ Granda Ospedale Maggiore Policlinico, Milano 20122, Italy; stefano.gatti@unimi.it

**Keywords:** NAFLD, fibrosis, HCC, hepatic stellate cells, miR-101-3p

## Abstract

Insulin resistance (IR) and microRNAs (miRNAs), which regulate cell-to-cell communication between hepatocytes and hepatic stellate cells (HSCs), may intertwine in nonalcoholic fatty liver disease (NAFLD) pathogenesis. The aim of this study was to evaluate whether epigenetics and environmental factors interact to promote progressive NAFLD during IR. We examined the miRNA signature in insulin receptor haploinsufficient (InsR+/−) and wild-type (wt) HSCs by RNAseq (*n* = 4 per group). Then, we evaluated their impact in an IR-NASH (nonalcoholic steatohepatitis) model (InsR+/− mice fed standard or methionine choline deficient (MCD) diet, *n* = 10 per group) and in vitro. InsR+/− HSCs displayed 36 differentially expressed miRNAs (*p* < 0.05 vs. wt), whose expression was then analyzed in the liver of InsR+/− mice fed an MCD diet. We found that miR-101-3p negatively associated with both InsR+/− genotype and MCD (*p* < 0.05) and the histological spectrum of liver damage (*p* < 0.01). miR-101-3p was reduced in InsR+/− hepatocytes and HSCs and even more in InsR+/− cells exposed to insulin (0.33 µM) and fatty acids (0.25 mM), resembling the IR-NASH model. Conversely, insulin induced miR-101-3p expression in wt cells but not in InsR+/− ones (*p* < 0.05). In conclusion, IR combined with diet-induced liver injury favors miR-101-3p downregulation, which may promote progressive NAFLD through HSC and hepatocyte transdifferentiation and proliferation.

## 1. Introduction

Nonalcoholic fatty liver disease (NAFLD) represents the leading cause of progressive liver disorders worldwide, affecting between 20% to 40% of the adult population [1]. NAFLD is defined by increased hepatic fat accumulation not explained by alcohol abuse, and it encompasses a wide spectrum of liver injuries, ranging from simple steatosis (>5% of liver weight) to its inflammatory form nonalcoholic steatohepatitis (NASH). NASH may be complicated by the presence of fibrosis and, eventually, it may progress to cirrhosis and hepatocellular carcinoma (HCC) [2,3]. NAFLD has a strong inherited component, and variants in proteins regulating hepatocellular lipid handling, including patatin-like phospholipase domain-containing 3 (PNPLA3), transmembrane 6 superfamily member 2 (TM6SF2), membrane bound O-acyltransferase domain-containing 7 (MBOAT7), predispose to the disease development and progression to NASH and fibrosis [4]. Dietary habits such as excessive caloric intake, fructose consumption, and physical inactivity represent other risk factors for NAFLD development [5]. In addition, its pathogenesis is closely intertwined with excessive adiposity, insulin resistance (IR), and metabolic syndrome [6].

IR even correlates with the severity of liver fibrosis [7], the main determinant of NAFLD prognosis [5]. Indeed, fibrosis has been observed in diabetic patients with NAFLD even independently of NASH [6], and genetic variants that impair insulin receptor (InsR) signaling favor fibrosis development in NAFLD patients [8]. The mechanism whereby IR aggravates fibrosis may be related to reduced extracellular matrix (ECM) degradation and to the induction of proteins involved in collagen crosslinking [9].

A new perspective of NAFLD pathogenesis and management is provided by epigenetics, which modulate transcriptome in response to environmental cues. In particular, microRNAs (miRNAs) are short non-protein coding, single-strand RNAs of 19–22 nucleotides that regulate gene expression and cell-to-cell communication as it occurs between hepatocytes and hepatic stellate cells (HSCs) during fibrogenesis [10]. Alteration of miRNA expression in response to genetic/epigenetic factors or environmental conditions may contribute to steatosis onset and NAFLD progression to fibrosis and cancer [11], mirroring the histological features and the molecular events occurring in NAFLD [12]. Thus, they could be exploited as attractive candidate biomarkers for an accurate profiling of the different stages of liver injury, enabling early and non-invasive diagnosis and the clinical monitoring of the disease progression.

Therefore, we aimed to explore the mechanisms through which epigenetics and environmental factors interact to promote progressive NAFLD in the context of IR. To this purpose, we first assessed which miRNAs were deregulated by IR in HSCs whose activation represents the hallmark of liver fibrosis. Secondly, we evaluated whether the miRNAs differentially expressed in insulin resistant HSCs could impact on NAFLD-related fibrosis by using an experimental model of IR-NASH. Thus, to investigate the independent contribution of IR and NASH that is usually intertwined in patients on miRNAs expression, IR was induced in mice by haploinsufficiency for InsR (InsR+/−) [13,14,15], which determines an impairment of hepatic insulin signaling recapitulating that has been observed in NAFLD patients [8]. Conversely, to model fibrogenic NASH, we exploited the methionine choline deficient (MCD) diet, which impairs phospholipid metabolism without altering IR [16]. Finally, we tested the expression of the candidate miRNAs in immortalized human hepatoma cells (HepG2) and in HSCs (LX-2) in the presence or absence of miRNA mimics. Here, we showed that miR-101-3p was downregulated in InsR+/− HSCs and hepatocytes, its expression was reduced even in total livers of insulin resistant mice, and it was associated with all stages of liver disease.

Consistently with these results, target prediction analysis revealed that miR-101-3p is involved in insulin signaling, lipid metabolism, cell proliferation, fibrogenesis, and cancer susceptibility, possibly representing a molecular signature in NAFLD during IR. Furthermore, miR-101-3p overexpression in HepG2 and LX-2 reduced proliferation, migration, and invasiveness, regulating pro-fibrotic and pro-carcinogenic markers.

## 2. Materials and Methods

### 2.1. Experimental Models

C57Bl/6 mice (Charles River, Calco, Italy) were housed at constant room temperature (23 °C) under 12-hour light/dark cycles with ad libitum access to water in compliance with the Principles of Laboratory Animal Care (NIH publication 86-23). InsR+/− mice on C57Bl/6 background were kindly provided by Prof. Accili D., Columbia University, NY, USA [13]. Genotyping was performed using genomic DNA extracted from mouse tail [15]. Primers are listed in Table S1.

To induce NASH, wild-type (wt) and InsR+/− male mice (more susceptible to liver damage and fibrosis) were fed either MCD (14.8% protein, 12.4% fat, and 72.8% carbohydrate, Test Diet, London, United Kingdom) or regular chow (15.1% protein, 12.4% fat, and 72.4% carbohydrate, Test Diet, London, United Kingdom), from 6 weeks of age, for 6 weeks. We tested two timelines of MCD administration (3 and 6 weeks) in preliminary experiments by using a powder MCD, which induced a more severe liver damage compared to MCD pellet and early loss of 20% of body weight. Thus, to not run into ethical issues, we did not expose animals to a prolonged MCD regimen (8 weeks) but we selected the adequate timeline (6 weeks) that could be representative of both NASH and fibrosis. Experiments were conducted in 10 mice per group. Body weight was recorded weekly throughout the experiments. Before sacrifice, mice were fasted for 16 h and the interventions were done during the light cycle. All animal experiments were performed according to the experimental protocol approved by University of Milan and Italian Ministry of Health Review Boards (protocol 8/14). The biochemical and histological features of SD and MCD mice are provided in Table S2. Detailed methods and reagents are presented in the Supplementary Materials and Methods.

### 2.2. Isolation of Hepatic Stellate Cells

The detailed protocol and data analysis approach are described in the Supplementary Materials and Methods.

### 2.3. Histology and Immunohistochemistry

The detailed protocol and data analysis approach are described in the Supplementary Materials and Methods.

### 2.4. Transcriptome Analysis on Hepatic Stellate Cells (Discovery)

Isolated HSCs were cultured on plastic for three passages. Total RNA was isolated using miRNeasy micro-kit (Qiagen, Hulsterweg, Germany). The purity and the concentration of RNA samples were determined with the NanoDrop ND-1000 spectrophotometer (Thermofisher Scientific, Carlsbad, USA). The integrity of the RNA was assessed by electrophoresis and by RIN evaluation through Agilent 2100 Bioanalyzer (Thermofisher Scientific, Carlsbad, USA). RNA sequencing was performed in paired-end mode with a read length of 150 nt using the Illumina HiSeq 4000 (Novogene, Hong Kong, China). RNAseq mapping descriptive statistics, the detailed protocol, and data analysis approach are described in the Supplemental Materials and Methods and in Table S3.

### 2.5. Quantitative Real Time PCR (qRT-PCR) Validation of miRNAs in Total Livers

Custom Taqman Array Plates and qRT-PCR were used to confirm the expression profiles of miRNAs obtained from the RNAseq data and their potential biological relevance to disease pathogenesis. Total RNA was isolated using miRNeasy mini-kit (Qiagen, Hulsterweg, Germany) from total liver of InsR+/− and wt mice, fed a standard or MCD diet. Then, miRNAs were reverse transcribed using Taqman Advanced miRNA cDNA synthesis kit (Thermofisher Scientific, Carlsbad, U.S.A.), according to the manufacturer’s protocol. The relative gene expression was determined using QuantStudio 3 Real-Time PCR System and Taqman Fast Advanced Master Mix (Thermofisher Scientific, Carlsbad, USA). All miRNA expression was normalized by using a pool of endogenous housekeeping (hsa-miR-361-5p, hsa-miR-186-5p, hsa-miR-26a-5p, hsa-miR-191-5p, hsa-miR-451-5p, and hsa-miR-423-5p) and shown as the fold change, according to the 2^−ΔΔCt^ method. Primers used are listed in Table S4.

### 2.6. Bioinformatic Resources 

To clarify biological functions of the differential expressed miRNAs identified in HSCs and to investigate the pathways related to the miRNA-selected target genes, gene ontology (GO) enrichment analysis and Kyoto Encyclopedia of Genes and Genomes (KEGG) analysis were performed by DAVID (https://david.ncifcrf.gov, version 6.8) and Enrichr (http://amp.pharm.mssm.edu/Enrichr/enrich).

The miRNA-RNA target interaction analysis was evaluated using prediction software, including TargetScan (www.targetscan.org/) and miRanda (www.microrna.org/). GO analysis was also performed to explore the functional roles of miRNAs in terms of biological processes and molecular functions, and KEGG was used to determine the target genes in different biological pathways.

### 2.7. In Vitro Treatments 

HepG2 (obtained from ATCC, ATCC-HB-8065) and LX-2 (Sigma-Aldrich, St. Louis, United States, SCC064) cells were cultured in Dulbecco’s modified Eagle’s medium (DMEM; Gibco-ThermoFisher Scientific Waltham, United States), supplemented with 10% FBS, 2 mM L-glutamine, 100 units/ml penicillin, and 0.1 mg/ml streptomycin, and incubated in a 5% CO_2_ humidified incubator at 37 °C. HepG2 and LX-2 were exposed to 0.33 µM insulin (Insuman Rapid, Sanofi Aventis) for 6 h.

In another experimental setting, hsa-miR-101-3p mimic (# 4464066) or miR-1 positive control (# 4464062) (mirVana miRNA mimic, ThermoFisher Scientific) were administered at a final concentration of 25 nM in complete medium without antibiotics and 0.5% bovine serum albumin (BSA). The cells were washed with PBS and then transiently transfected with 25 nM mir-101 mimic or miR-1 positive control using Lipofectamine 3000 (Thermo-fisher Scientific) according to the manufacturer’s instructions. We tested two different miRNA mimic concentrations (25 and 50 nM) and reached a great efficiency of transfection with both concentrations. Thus, we chose the lower concentration (25 nM), which has been frequently used in various studies [17,18,19]. At least three independent experiments were performed.

### 2.8. Cell Proliferation Assay

HepG2 and LX-2 cells are broadly used to study hepatic metabolism and related disorders as models in vitro. To explore the impact of miR-101-3p on hepatic fibrosis and cell proliferation, we used immortalized cell lines because of their higher rate of proliferation and their ability to maintain cell-specific features.

HepG2 and LX-2 cells (2500 cells/well) were seeded in 96-well plate in quadruplicate and incubated under normal culture conditions for 24 h prior to transfection. Cell proliferation was measured using the CellTiter96-Aqueous One Solution Cell Proliferation Assay (MTS) kit (Promega Corporation, Fitchburg, USA) according to the manufacturer’s instructions. MTS reagent (20 µL/well) was added to the cells followed by incubation for 4 h in 5% CO_2_ humidified incubator at 37 °C, and the absorbance was measured at 490 nm, at 0, 24, 48, and 74 h after transfection. At least three independent experiments were carried out.

### 2.9. Wound Healing Assay In Vitro 

HepG2 and LX-2 cells were seeded in 6-well plate (800,000 cells/well) in duplicate and incubated for 24 h prior to transfection with mir-101 mimic or miR-1 positive control (25 nM). When the cells reached ~90% confluency, a 20 µL pipette tip was used to scrap across the confluent layer, followed by gently washing with PBS three times and then by the addition of culture medium containing 10% fetal bovine serum (FBS). An area surrounded the wound was photographically monitored for 2 days consecutively. Wound healing ability was determined by measuring the scraped area alteration because of cell migration using ImageJ software. At least three independent experiments were conducted.

### 2.10. Statistical Analysis

Results are shown as means ± SD. Statistical analysis was performed using JMP 14.0 (SAS, Cary, NC, USA) by using two-way analysis of variance (ANOVA) or chi-square test, where appropriate. For descriptive statistics, continuous variables will be described as mean and standard deviation or median and interquartile range for highly skewed biological variables (i.e., alanine and aspartate aminotransferases (AST, ALT)). Variables with skewed distributions were logarithmically transformed before analyses. Analyses were performed by fitting data to generalized linear regression models. General linear models were fit to examine continuous traits. Independent histological and biochemical variables associated with miRNA expression in mice fed standard or MCD diet were assessed by multivariate logistic regression analysis, and were adjusted for the confounders specified in the Results section. Correlations were assessed by bivariate analysis or multivariate analyses after adjustment for confounding factors. *p*-values < 0.05 (two-tailed) were considered significant.

## 3. Results

### 3.1. Hepatic Stellate Cells Isolated from InsR+/− Mice Show a Distinctive Pattern of miRNA Expression

**Figure 1:**
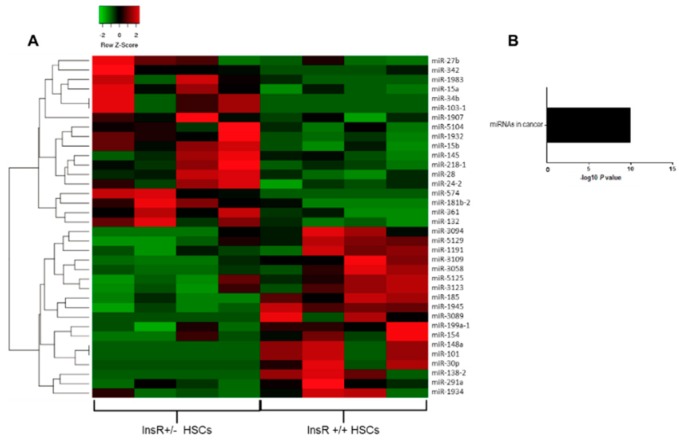
Hepatic stellate cells isolated from insulin receptor (InsR)+/− mice showed a distinctive patterns of microRNA (miRNA) expression. Heatmap was generated considering only specific miRNAs differentially expressed between InsR+/− and wild-type (wt) (InsR+/+) hepatic stellate cells (HSCs) (*n* = 4/group). We defined differentially expressed genes (DEGs), those with a *p*-value corrected (FDR) lower than 0.1 (as DESeq2 default) and with an absolute fold-change greater than 1.5. Red shading indicates induction and green shading indicates repression relative to the average levels of each miRNA across all samples (**A**). Kyoto Encyclopedia of Genes and Genomes (KEGG) pathways were enriched for differentially expressed miRNAs in InsR+/− compared to wt HSCs by RNA-seq analysis. The statistical significance level (*p*-value) was negative 10-based log trans-formed (**B**).

To investigate which miRNAs were deregulated in the context of IR, we isolated HSCs from wt and InsR+/− mice that showed an impairment in insulin signaling (Figure S1). Then, we performed a gene expression differential analysis of the whole transcriptome of InsR+/− vs. wt HSCs by RNAseq. We sequenced four replicates for each condition obtained from 40 to 61 million reads across the samples with an average percentage of uniquely mapped reads of 72% ± 11% (Table S3). We found 36 differentially expressed miRNAs, 18 being down-regulated and 18 over-expressed in InsR+/− cells (*p* < 0.05 vs. HSCs wt). Downregulated miRNAs included miR-1191, miR-3089, miR-101, miR-148a, miR-1945, miR-30p, miR-5125, miR-3109, miR-3123, miR-138-2, miR-3058, miR-199a-1, miR-185, miR-3094, miR-5129, miR-154, miR-1934, and miR-291a, whereas those upregulated were miR-103-1, miR-132, miR-15a/b, miR-181b-2, miR-1932, miR-218-1, miR-24-2, miR-34b, miR-28, miR-361, miR-574, miR-1983, miR-1907, miR-342, miR-5104, miR-27b, and miR-145 (Figure 1A). Pathway-enriched analysis revealed that deregulated miRNAs were involved in cellular response to endotoxins and molecules of bacterial origin (*p* = 2.8e^−5^; *q* * = 2.0e^−4^, * Benjamini-corrected vs. wt HSCs), response to oxygen levels (*p* = 4.5e^−10^; *q* * = 1.3e^−8^, *Benjamini-corrected vs. wt HSCs), positive regulation of angiogenesis and proliferation (*p* = 5.5e^−2^; *q* * = 1.6e^−1^, * Benjiamini-corrected vs. wt HSCs), and lipid metabolism (*p* = 9.6e^−3^; *q* * = 7.2e^−2^, * Benjiamini-corrected vs. wt HSCs).

As an independent approach, we also performed the enrichment analysis using KEGG gene sets, resulting in very similar processes, such as enrichment of miRNAs involved in proliferation and cancer (*p* = 7.1e^−11^; *q* * = 7.1e^−11^, * Benjiamini-corrected vs. wt HSCs), particularly in neoplastic cell transformation (Figure 1B). These data suggest that insulin-resistant HSCs displayed a peculiar pattern of miRNA expression that may predispose to HSCs proliferation, possibly inducing more advanced liver damage than their wt counterpart.

### 3.2. Differentially Expressed Hepatic miRNAs in InsR+/− Mice

To confirm the potential involvement of the differentially expressed miRNAs in IR-induced liver injury and to explore their impact on hepatic fibrosis, we also analyzed the expression of those miRNAs selected by RNAseq analysis to induce NASH in livers resected from InsR+/− and control mice challenged with regular or MCD diet for 6 weeks. Biochemical features of mice fed standard or MCD diet are shown in Table S2. We found that circulating alanine and aspartate aminotransferases (ALT, AST) were not increased in InsR+/− mice compared to wt fed an MCD diet, resembling what has been observed in IR- patients with type 2 diabetes (IR-T2D) with advanced fibrosis [20,21,22]. Despite similar body weight, fasting insulin and HOMA-IR were increased in InsR+/− mice, which also displayed enhanced hepatic Forkhead box protein O1 (FoxO1) protein expression, independently of diet administration [9]. In addition, InsR+/− mice fed an MCD diet exacerbated hepatic IR, further accumulating FoxO1 and impairing glucose metabolism. However, they preserved the activation of de novo lipogenesis and developed excessive hepatic fat accumulation, resembling the clinical situation observed in IR patients [9].

**Figure 2:**
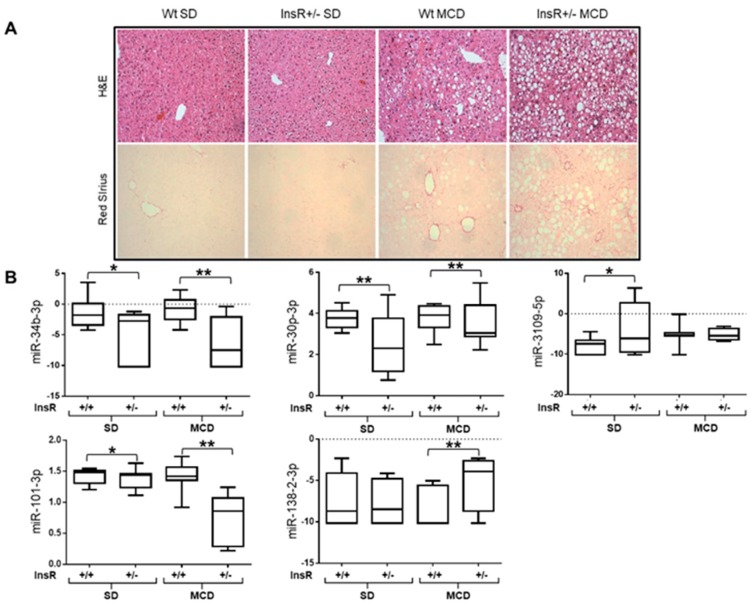
Differentially expressed hepatic miRNAs in InsR+/− mice. Hematoxylin and eosin (H&E) staining of hepatic specimens from wt and InsR+/− fed standard (SD) or methionine choline deficient (MCD) diet (n = 10 mice/group). Original magnification: 200× (A, upper panels). Hepatic Sirius Red staining in InsR+/− and wt mice fed an MCD diet for 6 weeks (**A**, lower panels). Hepatic miR-34b-3p, miR-30p-3p, miR-3109-5p, miR-101-3p, and miR-138-2-3p expressions were evaluated by qRT-PCR in lysates from total livers from wt (InsR+/+) and InsR+/− mice fed either an SD or MCD diet. Data were normalized for hsa-miR-361-5p, hsa-miR-186-5p, hsa-miR-26a-5p, hsa-miR-191-5p, hsa-miR-451-5p, and hsa-miR-423-5p average expression (n = 10 mice/group; * *p* < 0.05, ** *p* < 0.01) (**B**).

Indeed, InsR+/− mice fed an MCD developed a more severe hepatic steatosis and fibrosis (*p* < 0.01 vs. MCD diet-fed wt mice Figure 2A, Table S2), thus supporting the role of IR in NAFLD onset and in increasing fibrosis deposition [8].

Among the 36 miRNAs assessed, we found that miR-101-3p, miR-34b-3p, miR-30p-3p, miR-3109-5p, and miR-138-2-3p were differentially expressed in the liver of InsR+/− mice compared to wt (*p* < 0.05 vs wt). miR-101-3p, miR-34b-3p, and miR-30p-3p were downregulated in InsR+/− mice, whereas miR-3109-5p and miR-138-2-3p were upregulated (*p* < 0.01; *p* < 0.05; *p* = 0.07, respectively; Table 1). At the multivariate analysis, applying a generalized linear model, miR-34b-3p, miR-30p-3p, miR-3109-5p, and miR-101-3p were strongly associated with the InsR+/− genotype after the adjustment for the administration of the MCD diet, whereas the association between miR-138-2-3p and InsR+/− genotype was not significant (*p* = 0.07; Table 1).

Moreover, we also tested the interaction between MCD diet and InsR+/− genotype. miR-101-3p, miR-30p-3p, and miR-138-2 displayed an additive effect of MCD diet and InsR+/− genotype (*p* < 0.05, Figure 2B, Table 1). Specifically, miR-101-3p showed the stricter correlation to MCD and InsR+/− genotype and the additive effect was even more significant (Table 1).

Then, we assessed the association between the histological features of liver damage evaluated by using Kleiner score and the hepatic miRNA expressions. In the multivariate generalized linear model analysis comparing wt and InsR+/− mice fed a standard or MCD diet, we found a significant negative correlation between miR-34b-3p expression and the presence of histological steatosis (beta: −2.20; CI: −3.71 to −0.66; *p* = 0.006), lobular inflammation (beta: −2.50; CI: −4.30 to −0.66; *p* =0.009), and NAS score (beta: −1.41; CI: −2.30 to −0.51; *p* = 0.003), suggesting that its downregulation is involved in early stages of NAFLD (Table 2). Conversely, miR-138-2-3p was only associated with the end stages of liver disease, showing a positive correlation with the presence of fibrosis (beta: 1.37; CI: −0.03 to 2.77; *p* = 0.05), as shown in Table 2. Consistently, miR-34b-3p was inversely correlated with intrahepatic triglyceride content (*p* = 0.02; Figure S2A), supporting its steatogenic role, whereas miR-138-2-3p was positively correlated with hydroxyproline content (*p* = 0.001; Figure S2B) and Red Sirius positive areas (*p* = 0.03; Figure S2C), thus confirming its involvement in fibrosis development.

**Figure 3:**
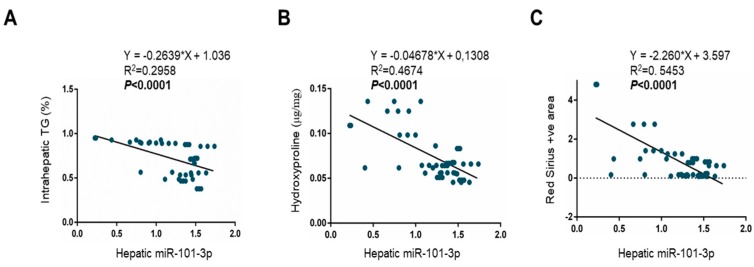
Hepatic miR-101-3p downregulation was related to all spectra of liver damage. Correlation analyses between miR-101-3p expression and intrahepatic triglyceride (TG) content (%) (**A**), hydroxyproline (μg/mg) concentration, representative of collagen deposition (**B**), and Red Sirius positive area, quantified by ImageJ software in 10 random non-overlapping fields for each mouse (**C**). miR-101-3p expression was evaluated by qRT-PCR in lysates from total livers from wt (InsR+/+) and InsR+/− mice fed either an SD or MCD diet (*n* = 10 mice/group). Data were normalized for hsa-miR-361-5p, hsa-miR-186-5p, hsa-miR-26a-5p, hsa-miR-191-5p, hsa-miR-451-5p, and hsa-miR-423-5p average expression.

Interestingly, only miR-101-3p was found to be significantly related to all spectra of liver damage from steatosis (beta: −0.16; CI: −0.33 to 0.001; *p* = 0.05), to lobular inflammation (beta: −0.21; CI: −0.41 to −0.02; *p* = 0.03), and fibrosis (beta: −0.30; CI: −0.44 to −0.15; *p* = 0.0001) (Table 2). Indeed, miR-101-3p showed a significant trend with all parameters analyzed, negatively correlating with intrahepatic triglycerides (*p* < 0.0001; Figure 3A), hydroxyproline concentration (*p* < 0.0001; Figure 3B), and Red Sirius positive areas (*p* < 0.0001; Figure 3C).

With regard to miR-30p-3p and miR-3109-5p, we did not find any association between them and the histological features.

Overall, these data suggest that miR-34b-3p, miR-138-2-3p, and miR-101-3p could specifically profile the stage of liver injury related to IR. In particular, miR-101-3p exhibited the strongest interaction between MCD and IR, showing a lower expression during the co-existence of genetic IR and diet-induced liver damage.

### 3.3. Pathway-Enriched Analysis of Differentially Expressed miRNAs in HSCs

Bioinformatic analysis was performed to gain insight into the functional impact of targeted genes of miR-101-3p. By using the software TargetScan, we obtained a list of the predicted targets that may be potentially involved in metabolic diseases and liver damage in the context of IR.

**Figure 4:**
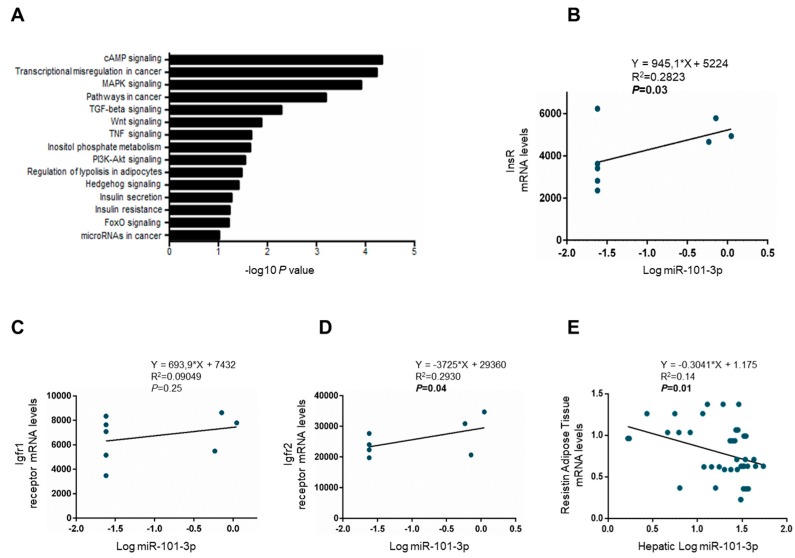
Hepatic stellate cells (HSCs) and hepatic miR-101-3p downregulation contributed to IR. KEGG pathways enriched for miR-101-3p targeted genes by DAVID. The statistical significance level (p-value) was negative 10-based log trans-formed (**A**). Correlation analyses between miR-101-3p expression evaluated by RNAseq in wt and InsR+/− HSCs and insulin receptor (InsR) expression (**B**), insulin-like growth factor 1 receptor (Igfr1) (**C**), and Igfr2 (**D**). Correlation analyses between miR-101-3p expression assessed by qRT-PCR in total livers from wt and InsR+/− mice fed either standard (SD) or MCD diets (n = 10 mice/group) and adipose tissue expression of resistin. Resistin mRNA levels were evaluated by qRT-PCR and normalized for beta-actin as housekeeping (n = 10/group) (**E**).

Analyzing miR-101-3p targets, we obtained a total of 720 genes from predicted databases involved in cellular response to insulin stimulus, insulin signaling activation, inositol phosphate metabolism, insulin secretion, IR, and regulation of lipolysis in adipocytes, as well as ECM organization, epithelial to mesenchymal transition, cell adhesion, proliferation, and differentiation. The data obtained from GO analysis is summarized in Table S5, and the most significantly enriched biological processes were exhibited according to the count of genes involved. Pathways from KEGG were significantly enriched in insulin signaling activation, inositol metabolism, and cancer development (Figure 4A; Table S6). These findings further reinforced the results observed in our model of IR-NASH, supporting the implication of miR-101-3p downregulation in the development and worsening of liver damage aggravated by the presence of genetic IR.

Then, we focused on genes involved in insulin signaling and we found that miR-101-3p expression positively correlated with mRNA levels of InsR and insulin-like growth factor receptor 1 and 2 (Igfr1/2) in HSCs analyzed by RNAseq (*p* < 0.05; Figure 4B–D). Moreover, hepatic miR-101-3p expression was inversely related to the adipose tissue expression of Resistin in wt and InsR+/− mice fed either a standard or MCD diet (*p* < 0.05; Figure 4E). Indeed, lower levels of miR-101-3p were associated with higher resistin, an adipokine that promotes IR and inflammation at a hepatic level, secretion into the blood flow in wt and InsR+/− mice fed either a standard or MCD diet, suggesting that its alteration may contribute to IR by regulating adipose tissue-specific adipokines, (*p* < 0.05; Figure 4E).

### 3.4. miR-101-3p and Pathway-Related Targets Involved in Fibrosis and Cell Proliferation in HSCs

**Figure 5:**
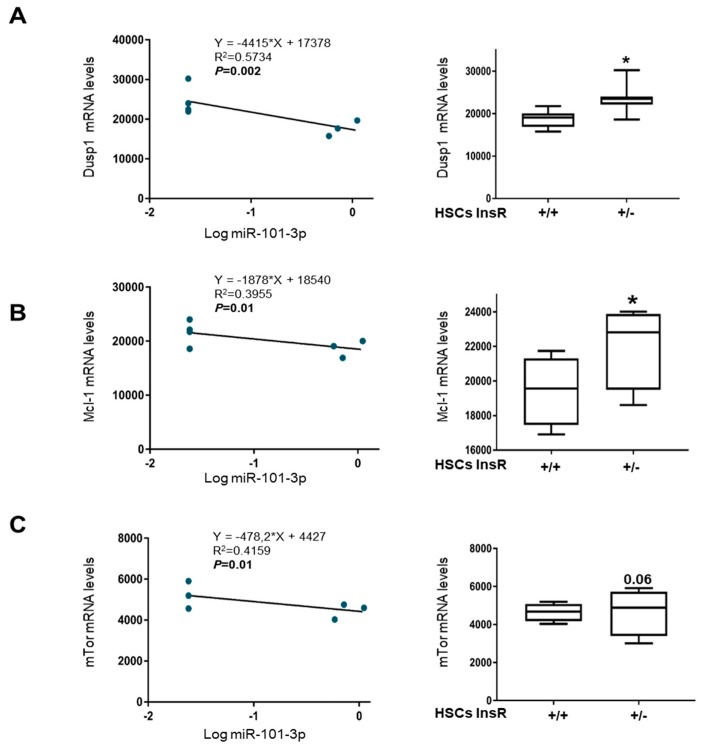
Predicted gene targets of miR101b-3p were up-regulated in InsR+/− HSCs. Correlation analyses between miR-101-3p analyzed in wt and InsR+/− HSCs and dual specificity protein phosphatase 1 (Dusp1). Dusp1 mRNA levels in wt and InsR+/− HSCs by RNAseq (**A**). Correlation analyses between miR-101-3p assessed in wt and InsR+/− HSCs and Mcl-1. Mcl-1 mRNA levels in wt and InsR+/− HSCs by RNAseq (**B**). Correlation analyses between miR-101-3p analyzed in wt and InsR+/− HSCs and mammalian target of rapamycin (mTor). mTor mRNA levels in wt and InsR+/− HSCs by RNAseq (**C**) (*n* = 4/group; * *p* < 0.05 vs. wt (InsR+/+) HSCs).

Since the hepatic expression of miR-101-3p was markedly decreased in InsR+/− mice fed MCD (Figure 2B), we next focused on better understanding its impact on fibrogenesis promotion and HCC development by evaluating the expression of miR-101-3p putative mRNA targets, previously predicted by bioinformatic analysis and proven by experimental evidence [14] in InsR+/− HSCs compared to wt by exploiting RNAseq data. As expected, markers of liver fibrosis, angiogenesis, proliferation, cell cycle progression, invasion, and migration such dual specificity protein phosphatase 1 (Dusp1), Mcl-1, mammalian target of rapamycin (mTor), vascular endothelial growth factor (Vegf-c), and c-Fos were inversely correlated with miR-101-3p levels in HSCs (*p* < 0.05; Figure 5A–C and Figure S3A,B), showing higher expression in HSCs InsR+/− compared to wt (*p* < 0.05 or *p* < 0.01 vs. wt HSCs; Figure 5A–C and Figure S3A,B). Furthermore, even zonula occludens-1 (ZO-1), which has cell adhesion properties, was positively correlated to the expression of miR-101-3p in HSCs (*p* < 0.01; Figure S3C) and it displayed lower levels in InsR+/− HSCs compared to wt ones (*p* < 0.01 vs. wt HSCs; Figure S3C), supporting the fact that the down-regulation of miR-101-3p observed during IR may predispose to advanced fibrosis and exert pro-carcinogenic effects.

**Figure 6:**
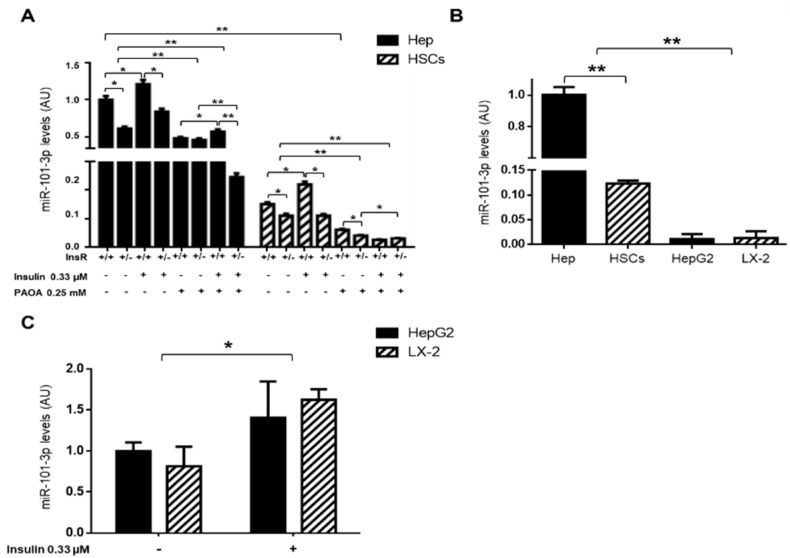
miR101b-3p expression was further reduced in in vitro models of IR-NASH and in high proliferating cells. Expression of miR-101-3p in wt and InsR+/− hepatocytes (Hep) and HSCs exposed to insulin (0.33 µM, for 6 h) alone or in a combination with palmitic (PA) and oleic (OA) acids (ratio 1:2, final concentration 0.25 mM, for 24 h) (**A**). Expression of miR-101-3p in primary mouse hepatocytes (Hep) and HSCs, and in human immortalized hepatoma cells (HepG2) and HSCs (LX-2) (**B**). Expression of miR-101-3p in HepG2 and LX-2, exposed to insulin (0.33 µM, for 6 h) (**C**). The expression was evaluated by qRT-PCR and normalized for hsa-miR-361-5p, hsa-miR-186-5p, hsa-miR-26a-5p, hsa-miR-191-5p, hsa-miR-451-5p, and hsa-miR-423-5p average expression and expressed as fold increase compared to untreated wt (InsR+/+) hepatocytes or HepG2 cells (arbitrary units—AU). At least three independent experiments were conducted (* *p* < 0.05, ** *p* < 0.01).

Finally, to confirm the pleiotropic downmodulation of miR-101-3p during IR, we isolated wt and InsR+/− primary mouse hepatocytes and HSCs. Next, we assessed miR-101-3p expression by qRT-PCR in the presence of a high concentration of insulin (0.33 µM) for 6 h and/or PAOA (0.25 mM) for 24 h, which mimicked hyperinsulinemia and diet-induced fat accumulation, respectively. We highlighted that miR-101-3p was expressed ~80% less in HSCs than hepatocytes (*p* < 0.01 vs. hepatocytes, Figure 6A) and that both insulin-resistant cell types displayed lower expression of miR-101-3p compared to their wt counterpart (*p* < 0.05 vs. wt hepatocytes or *p* < 0.05 vs. wt HSCs; Figure 6A). These data suggest that the ubiquitously reduction of miR-101-3p in IR may exert an impact on different cell types, possibly explaining its correlation with all spectra of liver damage.

In addition, insulin upregulated miR-101-3p in both wt hepatocytes and HSCs (*p* < 0.05 vs. untreated wt hepatocytes or HSCs; Figure 6A) and downregulated collagen (Col1A1) and α-sma mRNA levels—markers of fibrogenesis—in HSCs (*p* < 0.05 vs. untreated wt HSCs; Figure S4A). However, the effect of insulin on miR-101-3p was lost in InsR+/− HSCs (vs. untreated InsR+/− HSCs; Figure 6A), which in turn showed a higher expression of Col1A1 and α-sma (*p* < 0.05 vs. untreated InsR+/− HSCs; Figure S4A), further corroborating the hypothesis that miR-101-3p expression is mediated by the presence of the InsR. Indeed, the haploinsufficiency of InsR negatively regulated miR-101-3p expression, leading to fibrotic marker induction.

Conversely, the exposure to fatty acids dramatically hampered the expression of miR-101-3p in both wt and InsR+/− hepatocytes and HSCs (*p* < 0.01 vs. untreated wt hepatocytes and *p* < 0.01 vs. untreated wt HSCs; Figure 6A), reaching lower levels in InsR+/− cells co-stimulated with insulin and PAOA (*p* < 0.01 vs. InsR+/− PAOA hepatocytes or *p* < 0.05 vs. wt PAOA HSCs; Figure 6A).

It could be speculated that the marked downregulation of miR-101-3p observed in InsR+/− hepatocytes exposed to a combination of insulin and PAOA may resemble the decreased expression of this miRNA occurring in total liver in the context of IR and MCD interaction.

### 3.5. miR-101-3p was Down-Regulated in HepG2 and LX-2 Cells

Given the association between miR-101-3p and advanced liver injuries observed in our IR-NASH model, we evaluated the expression of miR-101-3p in immortalized cell lines by using hepatoma (HepG2) and hepatic stellates cells (LX-2), in vitro models commonly used to study liver fibrosis and cancer.

The expression of miR-101-3p was significantly downmodulated in both HepG2 and LX-2 compared to primary mouse hepatocytes and HSCs (*p* < 0.01 vs. primary cells; Figure 6B), thus representing a reliable model for studying the effects of miR-101-3p restoration in the presence of preserved insulin signaling. Moreover, in keeping with the previous results, the exposure to insulin (0.33 μM) increased the expression of miR-101-3p in both cell types (*p* < 0.05 vs. untreated cells; Figure 6C) and hampered, in turn, COL1A1 and α-SMA expression (*p* < 0.05 vs. untreated LX-2; Figure S4B), supporting the hypothesis that insulin signaling activation through insulin receptor may sustain miR-101-3p expression.

Therefore, we exploited these two cell lines characterized by rapid growth and low expression of miR-101-3p to further investigate the effect of miR-101-3p restoration on hepatocytes and HSC proliferation and invasiveness.

### 3.6. Overexpression of miR-101-3p Inhibited HepG2 and LX-2 Cell Proliferation and Migration

**Figure 7:**
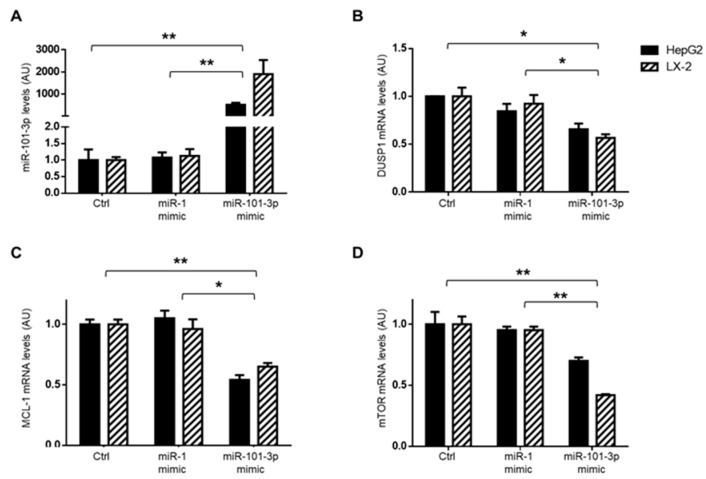
miR101b-3p mimic effectively increased miR-101-3p expression in HepG2 and LX-2 cells. Expression of miR-101-3p by qRT-PCR in HepG2 and LX-2 cells transfected for 24 h with 25 nM miR-101-3p mimic, 25 nM miR-1 mimic positive control (miR-1 mimic), or lipofectamine alone (Ctrl). Data were normalized for hsa-miR-361-5p, hsa-miR-186-5p, hsa-miR-26a-5p, hsa-miR-191-5p, hsa-miR-451-5p, and hsa-miR-423-5p average expression and expressed as fold increase compared to Ctrl (arbitrary units—AU). At least three independent experiments were conducted (* *p* < 0.05, ** *p* < 0.01) (**A**). DUSP1 (**B**), MCL-1 (**C**), and mTOR (**D**) mRNA levels were evaluated by qRT-PCR in HepG2 and LX-2 cells transfected for 24 h with 25 nM miR-101-3p mimic, 25 nM miR-1 mimic positive control (miR-1 mimic), or lipofectamine alone (Ctrl). Data were normalized for beta-actin as housekeeping. At least three independent experiments were conducted (* *p* < 0.05, ** *p* < 0.01).

**Figure 8:**
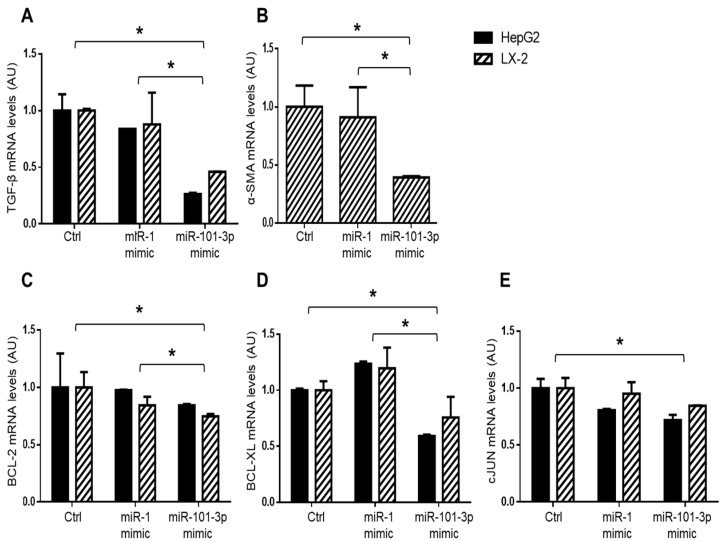
miR101b-3p mimic down-regulated genes involved in fibrosis and cell proliferation in HepG2 and LX-2 cells. TGF-β (**A**), α-SMA (**B**), BCL-2 (**C**), BCL-XL (**D**), and cJUN (**E**) mRNA levels were evaluated by qRT-PCR in HepG2 and LX-2 cells transfected for 24 h 25nM miR-101-3p mimic or 25nM miR-1 mimic positive control (miR-1 mimic) or lipofectamine alone (Ctrl). Data were normalized for beta-actin as housekeeping and are expressed as fold increase compared to Ctrl (arbitrary units—AU). At least three independent experiments were conducted (* *p* < 0.05, ** *p* < 0.01).

To deeply investigate the functional role of miR-101-3p in the promotion of liver fibrosis and hepatocarcinogenesis, we transfected HepG2 and LX-2 cell lines with 25 nM miR-101-3p mimic or with 25 nM miR-1 mimic positive control for 24 h. To evaluate the efficiency of our experimental setting, we first analyzed the expression of miR-1 as a positive control and found that miR-1-3p levels were dramatically increased in HepG2 and LX-2 cells (*p* < 0.01 vs. control and vs. miR-101-3p mimic; Figure S5A), as expected. Secondly, transient transfection with miR-101-3p mimic strongly enhanced the expression of miR-101-3p in both HepG2 and LX-2 cells, inducing a more than 500-fold higher expression of mature miR-101-3p compared to cells transfected with lipofectamine alone (negative control) or with miR-1 positive control (*p* < 0.01 vs. control and vs. miR-1 mimic, Figure 7A). Consistently, the expression of miR-101-3p target genes, DUSP1, MCL-1 and mTOR, was significantly downregulated in HepG2 and LX-2 cells treated with miR-101-3p mimic rather than negative controls (*p* < 0.05 or *p* < 0.01 vs. control; Figure 7B–D) or miR-1 positive control (*p* < 0.05 or *p* < 0.01 vs. miR-1 mimic; Figure 7B–D). Moreover, miR-101-3p induction hampered the expression of pro-fibrotic genes, such as TGF-β in both HepG2 and LX-2 cells (*p* < 0.05 vs. control and *p* < 0.05 vs. miR-1 mimic; Figure 8A) and α-SMA in LX-2 (*p* < 0.05 vs. control and vs. miR-1 mimic; Figure 8B), a marker of HSC activation. In addition, miR-101-3p overexpression markedly reduced BCL-2, BCL-XL and cJUN, which are markers of proliferation in both cell types (*p* < 0.05 vs. control and vs. miR-1 mimic; Figure 8C–E), thus supporting the ability of miR-101-3p to negatively regulate cell proliferation.

**Figure 9:**
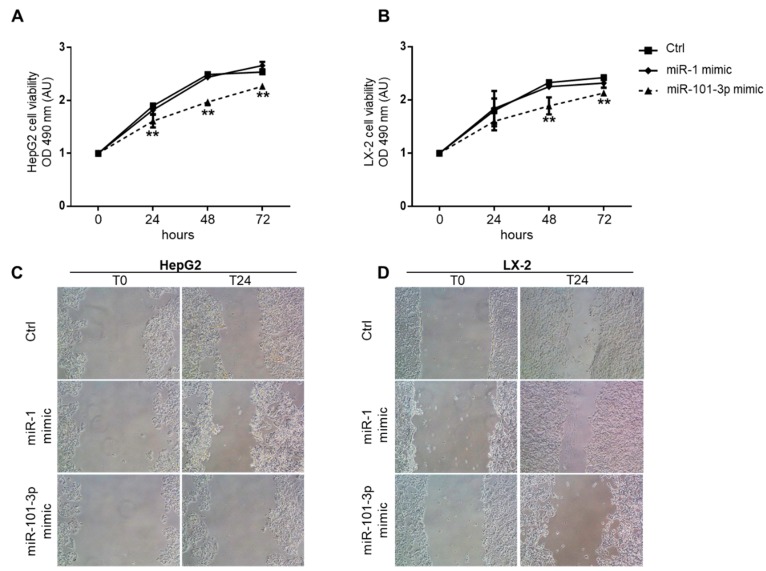
miR101b-3p mimic dampened proliferation and invasiveness of HepG2 and LX-2. Proliferations of HepG2 (**A**) and LX-2 (**B**) were assessed by MTS assays after transfection with 25 nM miR-101-3p mimic, 25nM miR-1 mimic positive control (miR-1 mimic), or lipofectamine alone (Ctrl). MTS absorbances at 490 nm were recorded at 0, 24, 48, and 72 h. Data are expressed as fold increase compared to Ctrl (arbitrary units—AU) (** *p* < 0.01 vs. Ctrl). Wound healing assay for HepG2 (**C**) and LX-2 (**D**). Representative images of the migrated cells were taken 0 and 24 h after the scratch formation. HepG2 and LX-2 were pre-treated with 25 nM miR-101-3p mimic, 25 nM miR-1 mimic positive control (miR-1 mimic), or lipofectamine alone (Ctrl) 24 h before the scratch. At least three independent experiments were conducted.

In line with the reduction of genes involved in cell cycle progression, the enforced expression of miR-101-3p significantly inhibited proliferation in HepG2 and LX-2 mimic transfection group compared to negative and positive miRNA mimic controls at 24, 48, and 72 h, as assessed using MTS assay (*p* < 0.01 vs. control and vs. miR-1 mimic; Figure 9A,B).

According to the reduced expression of miR-101-3p, even the low-proliferating primary InsR+/− HSC showed an enhanced rate of proliferation compared to wt forms at 48 and 72 h (*p* < 0.01 vs. wt HSCs; Figure S5B).

Finally, given the impact of cellular migration in liver fibrosis, cancer development, and metastasis, we next sought to evaluate the effect of miR-101-3p overexpression on the ability of migration of HepG2 and LX-2 cells by exploiting a wound-healing assay that revealed that miR-101-3p transfected cells healed the wound more slowly than controls (Figure 9C,D). Hence, we could speculate that miR-101-3p induction may contribute to prevent hepatic fibrosis development and progression to advanced stages of liver disease.

## 4. Discussion

In the present study, we explored the mechanisms through which epigenetics and environmental factors interact to promote progressive NAFLD in the context of IR. To this end, IR was induced in mice by haploinsufficiency for InsR (InsR+/−) [13,14,15], determining an impairment of hepatic insulin signaling that best recapitulates what is observed in NAFLD patients [8,23]. Conversely, to model fibrogenic NASH we exploited the MCD diet, which impairs phospholipid metabolism without altering IR [16]. This allowed the evaluation of the independent influence of IR and NASH on miRNA deregulation.

Therefore, we assessed the expression of miRNAs through different approaches, with the goal of studying the regulation of cell-to-cell communication between hepatocytes and HSCs that occurs during fibrogenesis, and to reveal candidate non-invasive biomarkers for an accurate profiling of the different stages of the liver injury, possibly enabling the early and non-invasive diagnosis and the clinical monitoring of the disease progression.

Firstly, we examined the miRNA signature in InsR+/− HSCs, which are characterized by an impaired insulin signaling, by analyzing the whole transcriptome, revealing 36 differentially expressed miRNAs compared to wt cells. These deregulated miRNAs were mainly involved in cell proliferation and cancer onset, probably profiling a peculiar pattern of miRNA expression that may orchestrate liver damage progression towards fibrosis and cancer development in IR conditions [4,24,25,26,27].

Secondly, we evaluated whether the miRNAs differentially expressed in insulin-resistant HSCs could impact on NAFLD-related fibrosis by using an experimental model of IR-NASH that develops hepatic IR reminiscent of that observed in patients with accumulation of FoxO1 and impairment of glucose metabolism but preserved activation of de novo lipogenesis [8,9]. This model does not completely reproduce the human NAFLD because the MCD diet induced weight loss and hypoglycemia, although it has been largely used to study intrahepatic events related to NASH because it replicates the histological spectrum of NAFLD from simple steatosis to advanced fibrosis [28]. However, as previously demonstrated by our group [8], this model of genetic IR was characterized by higher insulin levels and HOMA-IR, and was associated with increased steatosis and liver fibrosis accumulation, even independently of hepatic inflammation. Nonetheless, circulating transaminases were not increased in InsR+/− mice compared to wt fed an MCD diet resembling what has been observed in IR patients with type 2 diabetes (IR-T2D) and advanced fibrosis [20,21,22]. Indeed, it has been observed that a subgroup of type 2 diabetes (T2D) patients develop advanced fibrosis with normal enzymes even in the absence of NASH [20,21,22], thereby suggesting that IR can promote fibrosis deposition independently of inflammation, representing a complementary pathway, leading to advanced liver fibrosis. Moreover, genetic variants that impair InsR signaling favor fibrosis development in NAFLD [8]. We previously proposed that the mechanism whereby IR aggravates fibrosis seems to not be related to increased ECM deposition, but even more to reduced ECM turnover as highlighted by lower expression of metalloproteinases (MMPs) and enhanced production in enzymes involved in collagen crosslinking and pro-fibrogenic processes [9,29]. Therefore, we evaluated the hepatic expression of the 36 candidate miRNAs in InsR+/− mice fed MCD for 6 weeks and we demonstrated that miR-34b-3p, miR-138-2-3p, and miR-101-3p were strongly associated with InsR+/− genotype and MCD. Peculiarly, lower expression of miR-101-3p was closely associated with the co-existence of genetic IR and diet-induced liver damage, mirroring the mechanisms underlying epigenetic and environmental interplay. The main finding of this study is that among these three miRNAs, only the hepatic expression of miR-101-3p was related to all histological spectra of liver damage from steatosis to lobular inflammation and fibrosis in InsR+/− mice.

Pathway-enriched analyses of predicted target genes highlighted that miR-101-3p was mainly involved in metabolic processes such as inositol phosphate metabolism, insulin signaling activation, insulin secretion, IR, and regulation of lipolysis in adipocytes. According to these findings, in HSCs, miR-101-3p positively correlated with InsR and Igfr1/2. Furthermore, the reduced hepatic expression of miR-101-3p in InsR+/− mice influenced adipose tissue expression of resistin, an adipokine that promotes IR and propagates inflammation in the liver, thus contributing to IR through the regulation of key mediators of insulin signaling.

The link between miR-101 and insulin-related diseases has been reported in several preclinical and clinical studies. Indeed, miR-101 has been described in metabolic and autoimmune disorders, such as type 1 diabetes (T1D) and T2D, which have in common the loss of functional pancreatic β-cells. In particular, the pathogenesis of T1D is a complex mechanism involving the production of islet autoantibodies, genetics, and environmental factors. Zheng Y. and colleagues found that miR-101a is enriched in pancreatic cells and participates in β-cell dysfunction by decreasing the expression of antiapoptotic Bcl-2 and reducing pancreatic insulin secretion [30]. Higher circulating levels of miR-101-3p have also been reported to be associated with early-onset of T1D and T2D [31,32]. Therefore, the tissue expression of several miRNAs could be a consequence of a dynamic regulation of the rate of miRNAs produced and released into the circulation [4].

miR-101-3p was downmodulated in insulin-resistant primary hepatocytes and HSCs and even more in both InsR+/− cell types treated with insulin and fatty acid overload, which resembled what was observed in the total liver in the context of IR and MCD interaction. Conversely, insulin stimulated miR-101-3p expression in both wt hepatocytes and HSCs, but not in InsR+/− cells, suggesting that its expression was mediated by the presence of the insulin receptor. In line with miR-101-3p induction, collagen and α-sma levels were hampered in wt HSCs after insulin exposure. Conversely, markers of fibrogenesis were upregulated in InsR+/− HSCs, supporting that the impairment of insulin signaling may dampen miR-101-3p expression and favor fibrosis development. In addition, we confirmed this evidence also in HepG2 and LX-2 cells exposed to insulin. Similarly, even Zhu et al. demonstrated that inhibition of insulin signaling reduced miR-101 levels and enhanced its target genes in RAW264.7 macrophages [33].

Overall, our findings demonstrate that miR-101-3p downregulation was more strongly associated with hepatic injury when both IR and environmental stimuli, such as diet, coexisted in IR-NASH and in vitro models. Pathway analyses of predicted target genes also revealed an enrichment in endotoxins response, ECM deposition and organization, epithelial mesenchymal transition, cell adhesion, and TGF-β signaling, as well as cell proliferation and differentiation, possibly through Dusp1, mTOR, Mcl-1, and many others, as previously demonstrated [25,33,34]. Consistently, in insulin-resistant HSCs the expression of these genes was strongly enhanced, supporting the hypothesis that downregulation of miR-101-3p observed during IR may predispose to more advanced fibrosis and exert pro-carcinogenic effects. Indeed, several studies demonstrated that HSC activation and hepatic fibrogenesis is influenced by miR-101-3p downregulation, speculating that its restoration may ameliorate hepatic fibrosis [35]. Nevertheless, these studies showed that miR-101 was dampened in both hepatocytes and HSCs during CCl4-induced liver fibrosis as a consequence of transcriptional regulation of miR-101 by TGF-β signaling. The downregulation of miR-101 has been identified even in cardiac and pulmonary fibrosis [36,37]. Collectively, these findings strengthen the concept that deregulation of miR-101-3p is closely intertwined with fibrotic processes in different tissues.

Finally, to better explore the role of miR-101-3p in hepatic fibrosis and cell proliferation, we managed to overexpress it in human hepatoma cell lines (HepG2) and in HSCs (LX-2). We preferred to use immortalized cell lines because of their higher rate of proliferation and their ability to maintain cell-specific features. As rapidly proliferating cells, both HepG2 and LX-2 showed lower levels of miR-101-3p, as already reported by Hang Su et al. [38], compared with primary mouse hepatocytes and HSCs, thus representing a reliable model to study the effects of miR-101-3p restoration.

Notably, we demonstrated that miR-101-3p overexpression significantly inhibited the cell growth and exerted an impact on cellular migration and invasion in both hepatocytes and HSCs. Furthermore, the InsR+/− HSCs displayed a higher proliferation rate compared to the wt ones, further supporting the hypothesis that miR-101-3p downregulation promotes cell proliferation. In our study, the enhanced expression of miR-101-3p inhibited pro-fibrotic genes (TGF-β and α-SMA) and the anti-apoptotic genes BCL-2, BCL-XL, cJUN, DUSP1, MCL-1, and mTOR, possibly explaining the suppression of proliferation rate and the escape from apoptosis. miR-101 has been described as one of the most downregulated miRNAs in cancer-associated fibroblasts (CAFs), the main component of tumor stroma that creates a micro-environment that favors cancer progression [39]. In these cells isolated from lung tissue, the overexpression of miR-101 significantly impaired the ability of CAFs to stimulate tumor cell proliferation, migration and invasion, and enhanced apoptosis. Furthermore, loss of miR-101 promotes TGFβ1-induced epithelial mesenchymal transition process and the expression of mesenchymal markers in hepatocytes. Recently, miR-101-3p has been reported as a potent tumor suppressor in cancer development, progression, and therapy because it enhances doxorubicin-induced cytotoxicity and impacts upon chemoresistance of cisplatin in HepG2 cells [40,41,42]. According to our results, miR-101 downregulation was previously observed in multiple cancerous tissues, such as lung, colon, liver, gastric, breast, ovary, and prostate [25,43,44,45,46] decreasing early when tumors are poorly differentiated. Nonetheless, Li et al. [25] analyzed the data from The Cancer Genome Atlas (TCGA) and demonstrated that miR-101-1 expression was lower in cancer than in non-tumor liver tissues, and that this downregulation was also closely related to poor differentiation; high tumor, node, and metastasis (TNM) classification; poor tumor stage; positive lymph node metastasis; high alpha-fetoprotein; and poor overall survival in patients [47], assuming a great diagnostic and prognostic value of HCC [25]. Moreover, circulating miR-101 plasma levels were dramatically reduced in distant metastatic HCC patients and interestingly miRNA-based targeted therapy abrogated HCC growth in the liver, intrahepatic and distant metastasis to the lung, and mediastinum in an animal model of liver cancer [47].

## 5. Conclusions

In conclusion, all these findings indicate that IR is associated with a distinctive miRNA signature in both hepatocytes and HSCs, which may promote their proliferation and transdifferentiation. Specifically, miR-101-3p downregulation may profile all stages of liver disease, promoting the hepatic fibrosis in IR conditions by enhancing activation and survival of HSCs and predisposing to cancer development, inducing anti-apoptotic gene expression. Further studies are needed to deeply investigate the mechanisms linking IR and miR-101-3p in both early stages and progressive NAFLD with the goal of considering miR-101 as a candidate non-invasive biomarker to early diagnose hepatic fibrosis onset in NASH patients with IR.

## Figures and Tables

**Table 1:** Independent predictors of hepatic miRNA levels in wt and InsR+/− mice fed a standard or MCD diet.

miRNAs	InsR+/− Genotype			MCD Diet			Interaction (InsR+/− * MCD)		
	β	95% CI	p *	β	95% CI	p *	β	95% CI	p *
miR-34b-3p	−1.71	−2.54 to −0.89	0.0001	−0.23	−1.06 to 0.59	0.57	−0.41	−0.24 to 0.41	0.32
miR-101b-3p	0.13	−0.21 to −0.05	0.0009	−0.15	−0.23 to −0.07	0.0002	−0.15	−0.23 to −0.08	0.0001
miR-30p-3p	−0.28	−0.55 to −0.02	0.03	0.17	−0.09 to 0.44	0.19	0.33	0.06 to 0.59	0.01
miR-3109-5p	0.92	0.02 to 1.84	0.04	0.29	−0.61 to 1.20	0.51	−0.81	−1.71 to 0.09	0.07
miR-138-2-3p	0.68	−0.07 to 1.43	0.07	0.47	−0.28 to 1.22	0.22	0.91	0.15 to 1.66	0.02

* At multivariate generalized linear models adjusted for MCD diet or InsR+/− genotype shown in the table.

**Table 2:** Histological variables associated with hepatic miRNA expression at multivariate generalized linear model analysis in wt and InsR+/− mice fed a standard or MCD diet.

	Steatosis			Lobular Inflammation			Fibrosis			NAS		
	β	95% CI	p *	β	95% CI	p *	β	95% CI	p *	β	95% CI	p *
miR-34b-3p	−2.20	−3.71 to −0.66	0.006	−2.50	−4.30 to −0.66	0.009	−0.54	−2.13 to 1.03	0.49	−1.41	−2.30 to −0.51	0.003
miR-101-3p	−0.16	−0.33 to 0.001	0.05	−0.21	−0.41 to −0.02	0.03	−0.30	−0.44 to −0.15	0.0001	−0.12	−0.21 to −0.01	0.02
miR-30p-3p	−0.33	−0.86 to 0.19	0.22	0.26	−0.37 to 0.90	0.41	−0.19	−0.71 to 0.32	0.45	−0.05	−0.37 to 0.27	0.74
miR-3109-5p	0.23	−1.47 to 1.95	0.78	−0.52	−3.52 to 0.47	0.13	−0.16	−1.83 to 1.5	0.84	−0.30	−1.32 to 0.72	0.56
miR-138-2-3p	0.19	1.30 to 1.69	0.79	0.78	−0.98 to 2.54	0.34	1.37	−0.03 to 2.77	0.05	0.27	−0.62 to 1.16	0.55

* At multivariate generalized linear models adjusted for the presence of MCD diet and InsR+/− genotype.

## Supplementary Materials

The following are available online at https://www.mdpi.com/2072-6643/11/11/2597/s1.
