# Supplementary material for: mir-101-3p Downregulation Promotes Fibrogenesis by Facilitating Hepatic Stellate Cell Transdifferentiation During Insulin Resistance
Source: Nutrients. 2019 Oct 29;11(11):2597. doi: 10.3390/nu11112597 (PMC6893471; doi:10.3390/nu11112597)
Supplement: Supplementary file 1 [file nutrients-11-02597-s001.zip › nutrients-613108-supplementary.docx]

**SUPPLEMENTAL MATERIALS AND METHODS**

*Chemicals*

Methionine and Choline deficient diet (MCD) was purchased from Test Diet (London, UK); insulin Insuman Rapid (0.75 U/Kg) was ordered from Sanofi Aventis (Milan, Italy). Dulbecco’s modified Eagle’s medium (DMEM), fetal bovine serum (FBS), phosphate-buffered saline (PBS), L-Glutamine, Penicillin/Streptomycin, Trypsin/EDTA and Hank’s balanced salt solution (HBSS) were obtained from Gibco-ThermoFisher scientific (Waltham, United States). Fast SYBR green master mix, hsa-miR-101-3p, hsa-miR-1-3p, miR-101-3p mimic and miR-1 mimic positive control was obtained from Life Technologies-ThermoFisher Scientific (Waltham, United States). Collagenase type IV from Clostridium Histolyticum, Pronase E from Streptomyces Griseus, DNAse I from bovine pancreas, Optiprep and Percoll density gradient media and fructose were purchased from Sigma-Aldrich (St. Louis, United States). Free fatty acids (FFAs) Quantification Kit, Triglyceride Quantification Kit and Hydroxyproline assay kit were purchased from BioVision (Milpitas, United States). Rat/Mouse Insulin ELISA kit (Millipore, United States) was bought from Merck Millipore (Billerica, United States).

*Samples collection and biochemical evaluation*

Blood samples were collected by tail vein puncture. Blood glucose was measured by an Accu-Check glucometer (Roche Diagnostics AVIVA, Mannheim, Germany) and fasting plasma insulin levels by ELISA (EZRMI-13K, Millipore, Billerica, MA). Plasma and hepatic triglycerides, total cholesterol, alanine aminotransferase (ALT) and aspartate aminotransferase (AST) levels were measured with an automated analyzer (CobasC 702, Roche, Switzerland). Hepatic triglycerides and circulating free fatty acids (FFAs) were measured by a colorimetric assay (BioVision, Milpitas, United States) according to the manufacturer’s instructions. At sacrifice, liver samples were rapidly removed, harvested and snap frozen in liquid nitrogen. Samples of liver were fixed in formalin and embedded in paraffin, for histological evaluation.

*Hepatic hydroxyproline content evaluation*

Liver samples (between 30 and 50 mg) were homogenized in water and hydrolyzed in 6 M HCl at 120 °C overnight. After centrifugation at 10’000 g for 3 minutes, 30 μl of samples were moved to a 96-well plate and oxidized with a solution containing 6% Chloramine T, for 5 minutes at room temperature. Thereafter, a solution with 4-dimethylamino-benzaldehyde (DMAB) and percloric acid/isopropanol was added. The final mixture was incubated at 60°C for 90 min and then the absorbance was determined at 560 nm, by the spectrophotometric method of Bergman and Loxley (Bergman and Loxley). Standard solutions containing 0 (blank), 0.2, 0.4, 0.6, 0.8, 1.0 μg/well of 4-hydroxy-L-proline were treated likewise. The standard curve was linear in this range (r = 0.98). The value of the liver hydroxyproline level was expressed as μg/mg of tissue.

*Isolation of hepatic stellate cells*

For transcriptome analysis, HSCs were isolated by a multi-step ethylene glycol tetra-acetic acid (EGTA)/pronase/collagenase perfusion technique, through microcannulation of portal vein to ensure efficient perfusion of tissue sample, as previously described [1].

-perfusion solution 1, based on Hanks solution (Life Technologies, Carlsbad, CA) and EGTA 0,5 mM (Sigma-Aldrich, St Louis, MO);

-perfusion solution 2 based on Hanks solution (Life Technologies, Carlsbad, CA) and 1 mg/mL pronase E (Roche, Basel, Switzerland), CaCl2 2 mM, HEPES 20 mM (Sigma-Aldrich, St Louis, MO)

-perfusion solution 3, made of Hanks solution with collagenase type IV (Sigma-Aldrich, St Louis, MO) 0,25 mg/ml, CaCl2 2 mM, HEPES 20 mM (Sigma-Aldrich, St Louis, MO).

All perfusion solutions were incubated at 37°C before use. Furthermore, we performed an *in vitro* digestion in a Dnase type I (Roche, Basel Switzerland) solution, composed of Dnase type I 0,2 mg/ml, CaCl2 2mM and 1% Pen/Strep.

Then, we separated HSCs, taking advantage of HSCs low density, due to a large amount of lipid droplets, since these cells store retinol as retinyl palmitate. For this purpose, we used a discontinuous density gradient of 15% and 11,5% Optiprep (Sigma-Aldrich, St Louis, MO), prepared according to the manufacturer's instruction.

HSCs were cultured on plastic in DMEM medium supplemented with 10% FBS, 2 mM L-glutamine, 100 units/ml penicillin and 0.1 mg/ml streptomycin. Isolated cells’ vitality was evaluated using flow cytometry and was around 99.5%.

*Isolation of hepatic cells*

To assess miR-101-3p in the hepatic cells, primary mouse hepatocytes and HSCs were isolated by a multi-step ethylene glycol tetra-acetic acid (EGTA)/collagenase perfusion technique, through microcannulation of portal vein to ensure efficient perfusion of tissue sample, as previously described [1].

Hepatocytes were cultured on plastic in DMEM medium supplemented with 10% FBS, 2 mM L-glutamine, 100 units/ml penicillin and 0.1 mg/ml streptomycin; 100 nM insulin and 100 nM dexamethasone. Isolated cells’ vitality was evaluated using flow cytometry and was around 99.5%. At least, three independent lots of freshly isolated cells were used for the experiments.

Hepatocytes and HSCs were treated with a combination of palmitic (PA) and oleic (OA) acids at the final concentration of 0.25mM (ratio 1:2) for 24 hours in presence or not of insulin (0.33 µM insulin; Insuman Rapid, Sanofi Aventis) for 6 hours.

*Histology and immunohistochemistry*

Liver tissue samples were fixed in 10% PBS buffered formalin. All tissues were embedded in paraffin within 24 hours of formalin fixation. Tissue sections were stained by Hematoxylin and Eosin (H&E) to assess liver pathology, according to Kleiner et al [2]. Liver fibrosis was revealed by Red Sirius staining. Collagen fibers deposition was quantified by ImageJ analysis software (https://imagej.nih.gov/ij/) in 10 random micrographs per each sample (magnification 200x) by calculating the Red Sirius positive area, as percentage of pixels above the threshold value with respect to the total pixels per area.

*Transcriptome analysis*

Raw reads were mapped against the Mouse Genome (mm9) using a custom pipeline based on the standard primary analysis procedure. The pipeline performed the primary analysis step including FASTQ quality check, low-quality reads trimming with Trimmomatic and the mapping procedure with STAR [3]. To select high-quality alignment (HQ-BAM) we filtered out the read with a mapping quality alignment (MAPQ) lower than 15. The pipeline produced a high-quality alignment file (BAM) for every sample. The transcript quantification step was performed with HTSeq. To remove possible batch effect due to library preparation and sequencing errors we corrected the read counts using RUVSeq. RNA-seq mapping descriptive statistics are shown in **Table S3**. The differential expression analysis was performed using DESeq2 R package. We defined differentially expressed genes (DEGs) those with a p-value corrected (FDR) lower than 0.1 (as DESeq2 default) and with an absolute fold-change greater than 1.5.

*Gene expression analysis*

miRNAs were isolated using miRNeasy mini-kit (Qiagen, Hulsterweg, Germany) from HepG2 and LX-2 cells. Then, miRNAs were reverse transcribed using Taqman Advanced miRNA cDNA synthesis kit (Thermofisher Scientific, Carlsbad, U.S.A.), according to the manufacturer's protocol. The relative gene expression was determined using QuantStudio 3 Real-Time PCR System and Taqman Fast Advanced Master Mix (Thermofisher Scientific, Carlsbad, U.S.A.). All miRNAs expression was normalized by using a pool of endogenous housekeeping (hsa-miR-361-5p, hsa-miR-186-5p, hsa-miR-26a-5p, hsa-miR-191-5p, hsa-miR-451-5p and hsa-miR-423-5p) and shown as the fold change, according to the 2-ΔΔCt method. Primers used were listed in **Table S7**.

Total RNA was extracted from tissues and cell cultures using Trizol reagent (Thermofisher Scientific, Carlsbad, U.S.A). Quantitative real time PCR (qRT-PCR) was performed by a QuantStudio 3 Real-Time PCR System (Life Technologies), using the SYBR Green chemistry (Fast SYBR Green Master Mix; Thermofisher Scientific, Carlsbad, U.S.A). All reactions were delivered in triplicate. Data were normalized to the β-actin gene expression and results were expressed as arbitrary units or fold increase as indicated in bar graphs. Primers are listed in **Table S8.**

*Western Blot Analysis*Total protein lysates were extracted from 20 mg of cell cultures, using RIPA buffer containing 1 mmol/L Na-orthovanadate, 200 mmol/L phenylmethyl sulfonyl fluoride and 0.02 μg/μL aprotinin. Samples were pooled prior electrophoretic separation and all reactions were performed in duplicate. Then equal amounts of proteins (50 μg) were separated by SDS-PAGE, transferred electrophoretically to nitrocellulose membrane (BioRad, Hercules, CA) and incubated with specific antibodies overnight. At least, three independent lots of freshly extracted proteins were used for experiments. Antibodies and concentration used are listed in **Table S9**.

**SUPPLEMENTARY TABLES**

**Table S1:** Genotyping primer sequences.

| **Gene** | **Sequence** |
| --- | --- |
| IR-S2 | AGCTGTGCACTTCCCTGCTCA |
| IR-AS2 | TCTTTGCCTGTGCTCCACTCT |
| IR-Neo3 | TTAAGGGCCAGCTCATTCCTC |

**Table S2:** Biochemical features and histological indices of liver damage of InsR+/+ and +/- mice upon SD or MCD feeding.

|  | | **InsR+/+ SD** | **InsR+/- SD** | **InsR+/+ MCD** | **InsR+/- MCD** | ***P*** |
| --- | --- | --- | --- | --- | --- | --- |
| Body Weight, g | 24.6±2.5 | 27.1±3.6 | 18.8±1.0 | 17.4±3.2 | **<0.0001** |  |
| Liver weight, g | 0.94±0.12 | 1.07±0.2 | 0.76±0.18 | 0.78±0.27 | **0.001** |  |
| Glucose, mg/dL | 130 ±19 | 133 ±23 | 77 ±24 | 88 ±16 | **<0.0001** |  |
| Insulin, ng/mL | 0.37±0.06 | 0.70±0.40 | 0.31±0.07 | 0.84±0.40 | **<0.0001** |  |
| HOMA-IR | 2.2±0.75 | 4.5±2.6 | 1.10±0.40 | 3.2±1.23 | **0.0001** |  |
| Total cholesterol, mg/dL | 69.4±7.8 | 75.6±6.6 | 36.6±17.3 | 26.3±2.7 | **<0.0001** |  |
| Triglycerides, mg/dL | 46.0±9 | 46.6±17.6 | 35.9±14 | 35.3±6.7 | 0.05 |  |
| FFAs, mmol | 0.9±0.18 | 0.51±0.10 | 0.20±0.17 | 0.29±0.15 | **<0.0001** |  |
| AST, IU/L | 160 [148-160] | 140 [122-184] | 297 [228-324] | 202 [112-238] | **<0.0001** |  |
| ALT, IU/L | 76 [65-84] | 52 [32-55] | 252 [246-490] | 105 [80-279] | **<0.0001** |  |
| Steatosis | NA | NA | 2.0±0.65 | 2.5±0.90 | **<0.0001** |  |
| Lobular inflammation | NA | NA | 2.4±0.5 | 2.8±0.45 | **<0.0001** |  |
| Ballooning | NA | NA | 0 | 0 | 1 |  |
| Portal inflammation | NA | NA | 1.4± 1.4 | 0.2 5±0.45 | **<0.0001** |  |
| Fibrosis | NA | NA | 1.4±0.5 | 1.8±0.9 | **<0.0001** |  |
| NAS | NA | NA | 4.4±1.0 | 5.25±1.36 | **<0.0001** |  |
| Intra-hepatic TG (%) | 4±0.7 | 3.94±0.9 | 5.7±2.5 | 8.30±0.5 | **<0.0001** |  |
| Red Sirius (% Area) | 0.15±0.04 | 0.11±0.04 | 1.2±0.80 | 2.10±1.63 | **<0.0001** |  |
| Hydroxyproline content | 0.06±0.01 | 0.06±0.01 | 0.07±0.03 | 0.11±0.03 | **<0.0001** |  |

Values are expressed as mean ± standard deviation (SD), median [interquartile range], or number (%). P values are referred to interaction between genotype and diet (generalized linear model).

AST: aspartate aminotransferase. ALT: alanine aminotransferase.

**Table S3:** RNA-seq mapping descriptive statistics.

|  | **InsR+/- HSCs** | **InsR+/+ HSCs** | **Total** |
| --- | --- | --- | --- |
| Raw reads (10^6^ reads) | 50±9 | 52±7 | 51±7 |
| Mapped reads (%) | 85±16 | 83±14 | 84±14 |
| Uniquely mapped (%) | 71±12 | 73±12 | 72±11 |
| Multi-Mapping reads (%) | 14±5 | 9±3 | 1c±4 |

Sequencing mapping statistics reporting the principal metrics aggregated by condition (4 samples each condition). The “Total” column reports the mean values across all the 8 analyzed samples. Values are expressed as mean ± standard deviation (SD).

**Table S4**. qRT-PCR miRNAs validated in total livers, by using Custom Taqman Array Plates.

|  | ***5p*** | ***3p*** |
| --- | --- | --- |
| miR-132 | mmu-miR-132-5p | mmu-miR-132-3p |
| miR-15a | mmu-miR-15a-5p | mmu-miR-15a-3p |
| miR-15b | mmu-miR-15b-5p | mmu-miR-15b-3p |
| miR-181b-2 | mmu-miR-181b-2 |  |
| miR-1932 | mmu-miR-1932 |  |
| miR-24-2 | mmu-miR-24-2-5p |  |
| miR-103-1 |  | mmu-miR-103-3p |
| miR-34b |  | mmu-miR-34b-3p |
| miR-361 | mmu-miR-361-5p | mmu-miR-361-3p |
| miR-574 | mmu-miR-574-5p | mmu-miR-574-3p |
| miR-28 | mmu-miR-28c | mmu-miR-28a-3p |
| miR-185 | mmu-miR-185-5p | mmu-miR-185-3p |
| miR-199a-1 |  | mmu-miR-199a-3p |
| miR-3058 | mmu-miR-3058-5p | mmu-miR-3058-3p |
| miR-3089 | mmu-miR-3089-5p | mmu-miR-3089-3p |
| miR-3094 | mmu-miR-3094-5p | mmu-miR-3094-3p |
| miR-30p |  | mmu-miR-30p-3p |
| miR-3109 | mmu-miR-3109-5p | mmu-miR-3109-3p |
| miR-5129 | mmu-miR-5129-5p | mmu-miR-5129-3p |
| miR-101-b | mmu-miR-101b-5p | mmu-miR-101-3p |
| miR-138 | mmu-miR-138-1-3p | mmu-miR-138-2-3p |
| miR-361 | hsa-miR-361-5p |  |
| miR-186 | hsa-miR-186-5p |  |
| miR-26a | hsa-miR-26a-5p |  |
| miR-191 | hsa-miR-191-5p |  |
| miR-451 | hsa-miR-451-5p |  |
| miR-423 | hsa-miR-423-5p |  |

**Table S5**. GO functional annotation for the most significantly enriched targeted genes of miR-101-3p

| **Biological process** | | **Count** | ***P*** | ***q*** |
| --- | --- | --- | --- | --- |
| Cell differentiation | 43 | 2,20e-3 | 1,10e-1 |  |
| Apoptotic process | 37 | 2,80e-4 | 2,80e-2 |  |
| Cell adhesion | 35 | 5,70e-5 | 7,40e-3 |  |
| Wnt signaling pathway | 18 | 1,00e-3 | 6,20e-2 |  |
| Cell proliferation | 14 | 4,70e-2 | 6,10e-1 |  |
| Vasculogenesis | 12 | 1,70e-5 | 3,00e-3 |  |
| Extracellular matrix organization | 11 | 5,40e-3 | 1,90e-1 |  |
| Notch signaling pathway | 11 | 1,00e-2 | 2,80e-1 |  |
| Cytokine-mediated signaling pathway | 11 | 2,70e-2 | 4,80e-1 |  |
| Regulation of growth | 9 | 2,30e-3 | 1,10e-1 |  |
| Liver development | 9 | 1,50e-2 | 3,60e-1 |  |
| Cellular response to insulin *stimulus* | 8 | 3,00e-2 | 4,90e-1 |  |
| Epithelial to mesenchymal transition | 7 | 5,30e-4 | 4,10e-2 |  |
| Response to endoplasmic reticulum stress | 7 | 4,40e-2 | 5,90e-1 |  |
| Fat cell differentiation | 7 | 5,20e-2 | 6,30e-1 |  |
| Cholesterol metabolic process | 7 | 8,20e-2 | 7,50e-1 |  |
| FGFR signaling pathway | 5 | 5,70e-2 | 6,60e-1 |  |
| Cellular response to cAMP | 5 | 8,90e-2 | 7,70e-1 |  |
| Positive regulation of TGFβR1 signaling pathway | 4 | 4,60e-2 | 6,10e-1 |  |
| TGFβR1 complex assembly | 2 | 9,80e-2 | 7,90e-1 |  |

Annotation: GO, Gene Ontology; Count, number of genes enriched in each GO term. *q* Benjamini adjusted p value.

**Table S6**. KEGG pathways enriched for miR-101-3p targeted genes by DAVID

| **KEGG Term** | | **Count** | ***P*** | ***q*** |
| --- | --- | --- | --- | --- |
| Pathways in cancer | 26 | 6,30E-04 | 2,20E-02 |  |
| MAPK signaling pathway | 21 | 1,20E-04 | 8,10E-03 |  |
| PI3K-Akt signaling pathway | 19 | 2,80E-02 | 1,70E-01 |  |
| cAMP signaling pathway | 19 | 4,50E-05 | 9,20E-03 |  |
| Transcriptional misregulation in cancer | 17 | 5,80E-05 | 6,00E-03 |  |
| MicroRNAs in cancer | 14 | 9,50E+02 | 3,10E-01 |  |
| Wnt signaling pathway | 11 | 1,30E-02 | 1,10E-01 |  |
| FoxO signaling pathway | 9 | 6,00E-02 | 2,30E-01 |  |
| TNF signaling pathway | 9 | 2,10E-02 | 1,50E-01 |  |
| TGFβ signaling pathway | 9 | 5,10E-03 | 5,40E-02 |  |
| Insulin resistance | 8 | 5,80E-02 | 2,30E-01 |  |
| Insulin secretion | 7 | 5,30E-02 | 2,30E-01 |  |
| Inositol phosphate metabolism | 7 | 2,20E-02 | 1,50E-01 |  |
| Regulation of lipolysis in adipocytes | 6 | 3,30E-02 | 1,80E-01 |  |
| Hedgehog signaling pathway | 4 | 3,80E-02 | 2,00E-01 |  |

Annotation: KEGG, Kyoto Encyclopedia of Genes and Genomes; Count, number of genes enriched in each KEGG term.

**Table S7**. qRT-PCR human miRNAs evaluated in HepG2 and LX-2.

|  | ***5p*** | ***3p*** |
| --- | --- | --- |
| miR-1 |  | hsa-miR-1-3p |
| miR-101 |  | hsa-miR-101-3p |
| miR-361 | hsa-miR-361-5p |  |
| miR-186 | hsa-miR-186-5p |  |
| miR-26a | hsa-miR-26a-5p |  |
| miR-191 | hsa-miR-191-5p |  |
| miR-451 | hsa-miR-451-5p |  |
| miR-423 | hsa-miR-423-5p |  |

**Table S8**. Quantitative PCR analysis human and mouse primer sequences.

|  | | ***Sense*** | | ***Antisense*** |
| --- | --- | --- | --- | --- |
| **α-SMA** | | TTCAATGTCCCAGCCATGTA | | GAAGGAATAGCCACGCTCAG |
| **α-sma mouse** | | GGAAAAGATCTGGCACCACT | | GAGTCCAGCACAATACCAGTTG |
| **β-ACTIN** | | GGCATCCTCACCCTGAAGTA | | GGGGTGTTGAAGGTCTCAAA |
| **β-ACTIN mouse** | | GCTACAGCTTCACCACCACA | | AAGGAAGGCTGGAAAAGAGC |
| **BCL-2** | | GAGGATTGTGGCCTTCTTTG | | ACAGTTCCACAAAGGCATCC |
| **BCL-XL** | | CGGTACCGGCGGGCATTCAG | | CGGCTCTCGGCTGCTGCATT |
| **COL1A1** | | GACTGGCAACCTCAAGAAGG | | CAATATCCAAGGGAGCCACA |
| **Col1a1 mouse** | | GAGCGGAGAGTACTGATCG | | TACTCGAACGGGAATCCATC |
| **DUSP1** | | CCTGACAGCGCGGAATCT | | GATTTCCACCGGGCCAC |
| **cJUN** | | ATCAAGGCGGAGAGGAAGCG | | TGAGCATGTTGGCCGTGGAC |
| **MCL-1** | | CCAAGAAAGCTGCATCGAACCAT | | CAGCACATTCCTGATGCCACCT |
| **mTOR** | | AGTGGACCAGTGGAAACAGG | | CAGTTCAGACCAGCAGGACA |
| **TGF-β** | | ACCCACAACGAAATCTATGACA | | CCCTCAATTTCCCCTCCAC |
| **Resistin mouse** | | TCCTTGTCCCTGAACTGCTG | | AAGACTGCTGTGCCTTCTGG |
|  |  | |  | |
|  |  | |  | |
|  |  | |  | |
|  |  | |  | |
|  |  | |  | |
|  |  | |  | |
|  |  | |  | |

**Table S9.** Antibodies used in Western blotting and catalog numbers.

| ***Antibody*** | | ***Catalog Number*** |
| --- | --- | --- |
| **Insulin Receptor (InsR) β (4B8)** | | Cell signaling #3025S |
| **Phospho (Tyr458)-PI3K p85/p55 (Tyr199)** | | Cell signaling #4228S |
| **PI3K p85** | | Cell signaling #4292S |
| **Phospho (Ser256)-FoxO1** | | Cell signaling #9461S |
| **FoxO1** | | Cell signaling #2880S |
| **α-sma** | | Abcam #ab5694 |
| **Tubulin** | | Abcam #ab6046 |
|  |  | |

**SUPPLEMENTARY REFERENCES**

1. Mederacke, I.; Dapito, D.H.; Affo, S.; Uchinami, H.; Schwabe, R.F. High-yield and high-purity isolation of hepatic stellate cells from normal and fibrotic mouse livers. *Nature protocols.* **2015**;*10(2)*:305-315.

2. Kleiner, D.E.; Brunt, E.M.; Van Natta, M.; Behling, C.; Contos, M.J.; Cummings O.W.; et al. Design and validation of a histological scoring system for nonalcoholic fatty liver disease. *Hepatology.* **2005**;*41(6)*:1313-21.

3. Dobin, A.; Davis, C.A.; Schlesinger, F.; Drenkow, J.; Zaleski, C.; Jha, S.; et al. STAR: ultrafast universal RNA-seq aligner. *Bioinformatics* (Oxford, England). **2013**;*29(1)*:15-21.

**Supplementary Figure Legends:**

**Figure S1:** Western blot analysis of proteins involved in insulin signaling in wt and InsR+/- primary mouse HSCs (InsR, phospho-phosphatidylinositol-3-kinase (p-PI3K), PI3K, phosho-Ser256-FoxO1, total FoxO1) and markers of fibrosis (α-sma). Tubulin has been used as housekeeping protein. Samples were pooled prior electrophoretic separation and all reactions were performed in duplicate. At least, three independent lots of freshly extracted proteins were used for experiments.

**Figure S2:** Correlation analyses between miR-34b-3p expression assessed in total livers from wt (InsR+/+)

and InsR+/- mice fed either standard (SD) or MCD (n=10 mice/group) and intrahepatic triglycerides (TG) content (%) (**A**). Correlation analyses between miR-138-2-3p expression assessed in total livers from wt (InsR+/+) and InsR+/- mice fed either standard or MCD (n=10 mice/group) and Hydroxyproline (μg/mg) concentration, representative of collagen deposition (**B**) and Red Sirius +ve area, quantified by ImageJ software in 10 random non-overlapping fields for each mouse (**C**). Hepatic miR-34b-3p and miR-138-2-3p expressions were evaluated by qRT-PCR in lysates from total livers from wt (InsR+/+) and InsR+/- mice fed either SD or MCD diet. Data were normalized for hsa-miR-361-5p, hsa-miR-186-5p, hsa-miR-26a-5p, hsa-miR-191-5p, hsa-miR-451-5p and hsa-miR-423-5p average expression.

**Figure S3:** Correlation analyses between miR-101-3p analyzed in wt (InsR+/+) and InsR+/- HSCs and Vegf-c. Vegf-c mRNA levels in wt (InsR+/+) and InsR+/- HSCs by RNAseq (**A**). Correlation analyses between miR-101-3p assessed in wt (InsR+/+) and InsR+/- HSCs and cFos. cFos mRNA levels in wt (InsR+/+) and InsR+/- HSCs by RNAseq (**B**). Correlation analyses between miR-101-3p analyzed in wt (InsR+/+) and InsR+/- HSCs and Zo-1 and Zo-1 mRNA levels in wt (InsR+/+) and InsR+/- HSCs by RNAseq (**C**) (n=4/group; *p<0.05 *vs* wt (InsR+/+) HSCs).

**Figure S4:** Expression of Col1a1 and α-sma by qRT-PCR in primary mouse wt and InsR+/- HSC before and after insulin 0.33 µM exposure for 6 hours (A). Expression of COL1A1 and α-SMA by qRT-PCR in LX-2 cells before and after insulin 0.33 µM exposure for 6 hours (B). mRNA levels were normalized to β-actin and expressed as fold increase (Arbitrary Units - AU) (*p<0.05, **p<0.01 *vs* untreated).

**Figure S5:** Expression of miR-1-3p by qRT-PCR in HepG2 and LX-2 cells transfected for 24 hours 25nM miR-101-3p mimic or 25nM miR-1 mimic positive control (miR-1 mimic) or lipofectamine alone (Ctrl). Data were normalized for hsa-miR-361-5p, hsa-miR-186-5p, hsa-miR-26a-5p, hsa-miR-191-5p, hsa-miR-451-5p and hsa-miR-423-5p average expression and expressed as fold increase compared to Ctrl (Arbitrary Units - AU). At least three independent experiments were conducted (*p<0.05, **p<0.01). (B) Proliferation of primary HSCs isolated from total livers of Wt and InsR+/- mice was assessed by MTS assay. MTS absorbances at 490nm were recorded at 0, 24, 48 and 72 hours. Data are expressed as fold increase compared to Ctrl (Arbitrary Units - AU) (**p<0.01 *vs* Wt HSCs).
