# Supplementary figures and images for: mir-101-3p Downregulation Promotes Fibrogenesis by Facilitating Hepatic Stellate Cell Transdifferentiation During Insulin Resistance
Source: Nutrients. 2019 Oct 29;11(11):2597. doi: 10.3390/nu11112597 (PMC6893471; doi:10.3390/nu11112597)

# Figure S1

Primary mouse HSCs

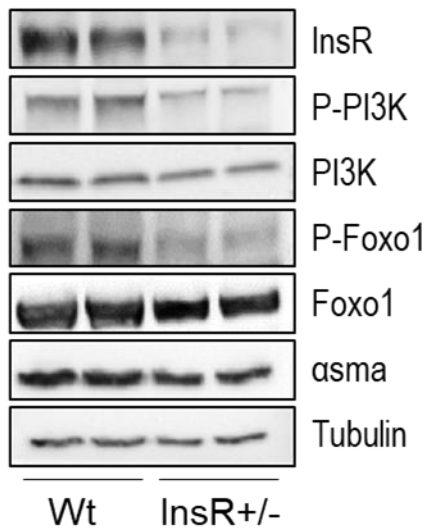

# Figure S2

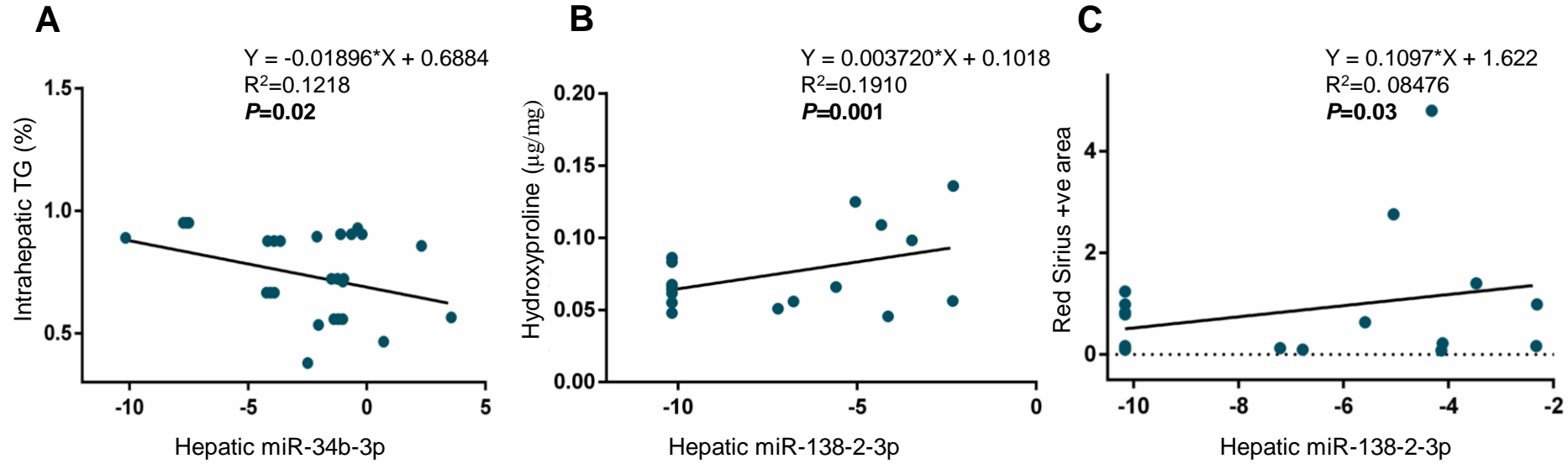

# Figure S3

**A**

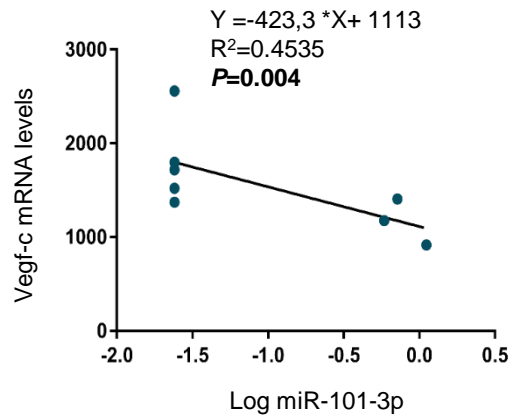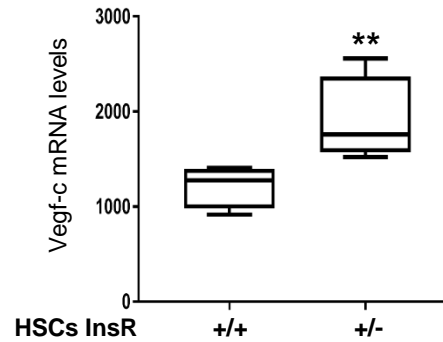

**B**

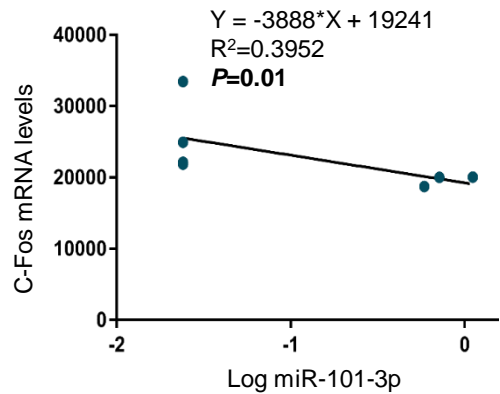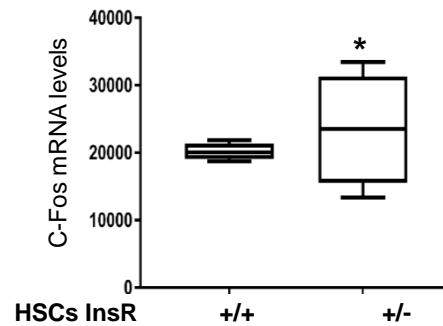

**C**

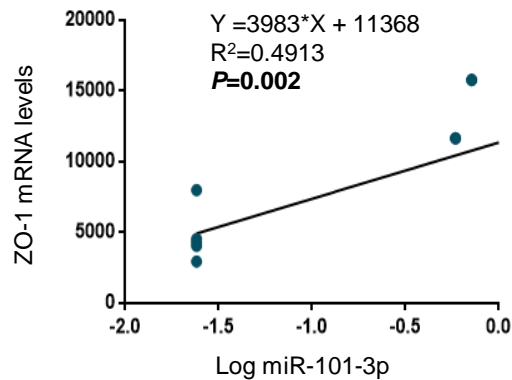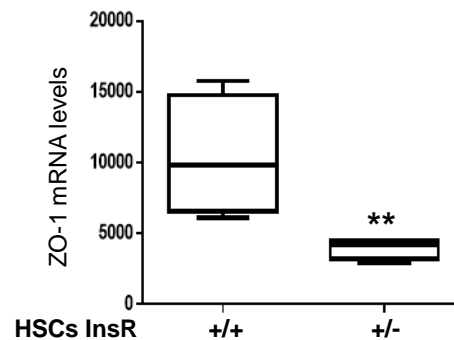

# Figure S4

**A**

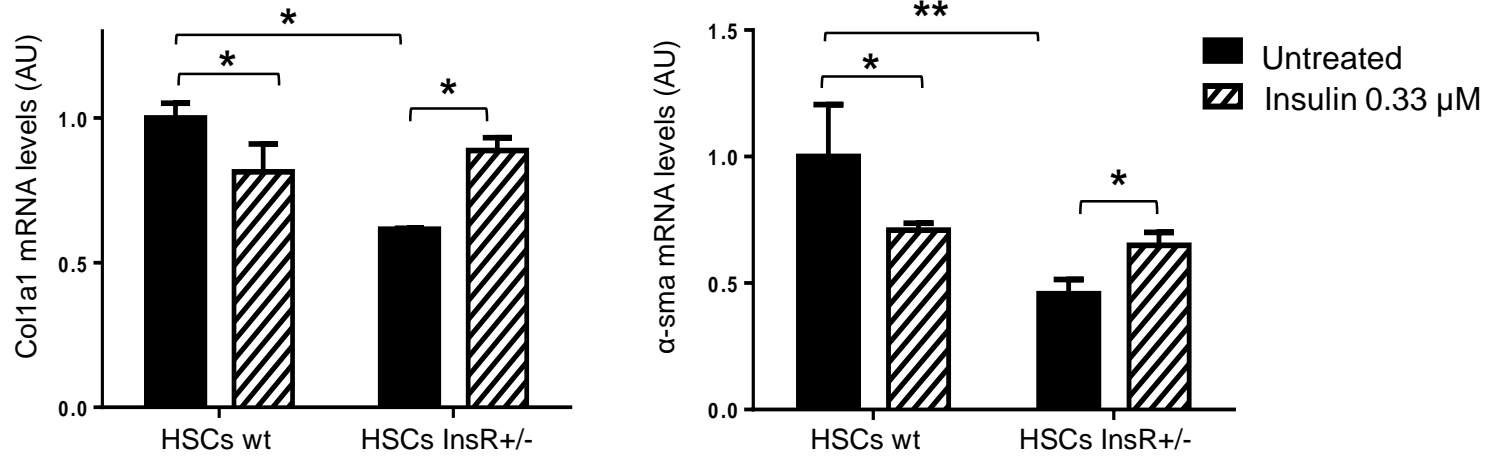

**B**

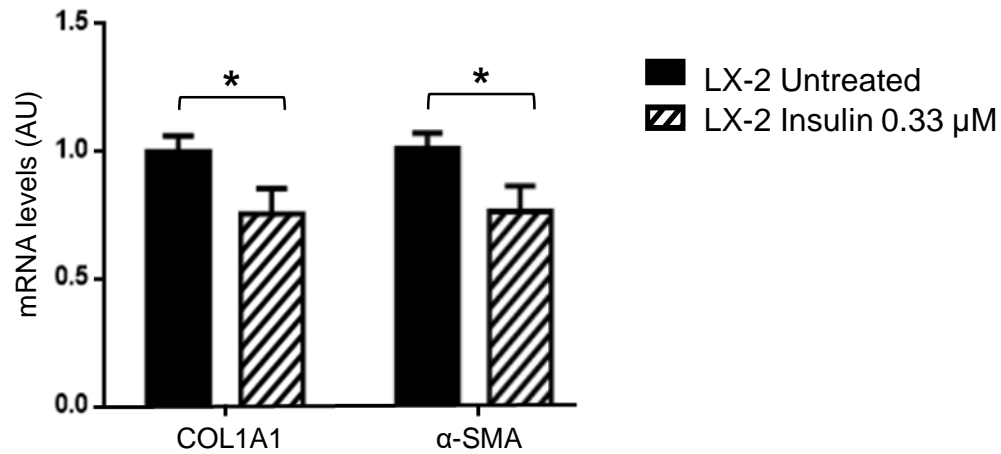

# Figure S5

## A

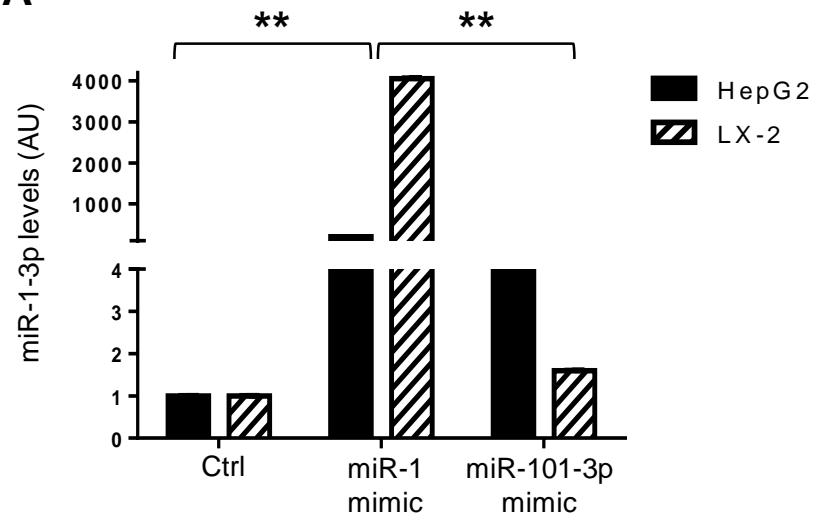

## B

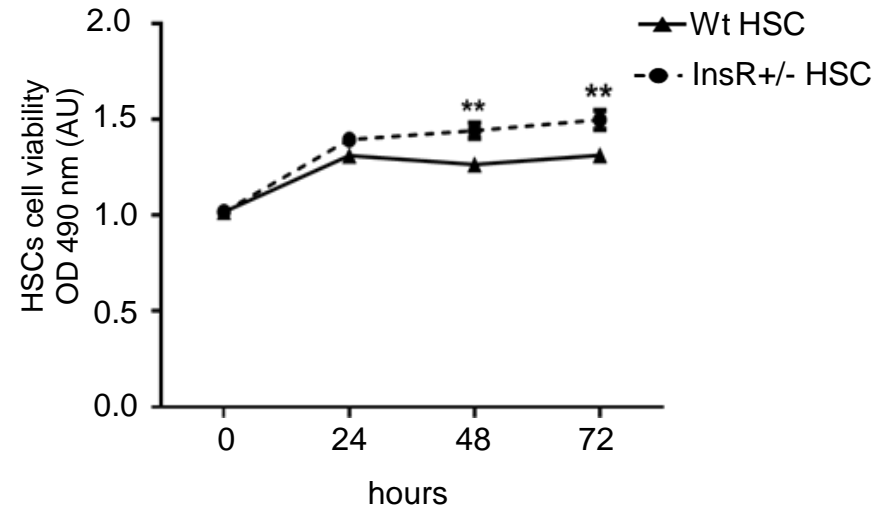

Supplement: Supplementary file 1 [file nutrients-11-02597-s001.zip › nutrients-613108-supplementary-figure.pdf]
